# A model for simulating emergent patterns of cities and roads on real-world landscapes

**DOI:** 10.1038/s41598-022-13758-1

**Published:** 2022-06-16

**Authors:** Takaaki Aoki, Naoya Fujiwara, Mark Fricker, Toshiyuki Nakagaki

**Affiliations:** 1 Faculty of Education, Kagawa University, Takamatsu, 760-8521 Japan; 2 Graduate School of Information Sciences, Tohoku University, Sendai, 980-8579 Japan; 3 PRESTO, Japan Science and Technology Agency, Kawaguchi, 332-0012 Japan; 4 Center for Spatial Information Science, The University of Tokyo, Kashiwa, 277-8568 Japan; 5 Institute of Industrial Science, The University of Tokyo, Tokyo, 153-8505 Japan; 6 Tough Cyberphysical AI Research Center, Tohoku University, Sendai, 980-8579 Japan; 7 Department of Plant Sciences, University of Oxford, Oxford, OX1 3RB UK; 8 Research Institute for Electronic Science, Hokkaido University, Sapporo, 001-0020 Japan; 9 Global Station for Soft Matter, Global Institution for Collaborative Research and Education, Hokkaido University, Sapporo, Japan

**Keywords:** Complex networks, Nonlinear phenomena

## Abstract

Emergence of cities and road networks have characterised human activity and movement over millennia. However, this anthropogenic infrastructure does not develop in isolation, but is deeply embedded in the natural landscape, which strongly influences the resultant spatial patterns. Nevertheless, the precise impact that landscape has on the location, size and connectivity of cities is a long-standing, unresolved problem. To address this issue, we incorporate high-resolution topographic maps into a Turing-like pattern forming system, in which local reinforcement rules result in co-evolving centres of population and transport networks. Using Italy as a case study, we show that the model constrained solely by topography results in an emergent spatial pattern that is consistent with Zipf’s Law and comparable to the census data. Thus, we infer the natural landscape may play a dominant role in establishing the baseline macro-scale population pattern, that is then modified by higher-level historical, socio-economic or cultural factors.

## Introduction

Across the surface of the earth throughout human history, civilisations have developed from small, scattered settlements into substantial agglomerations of towns and cities that are linked by increasingly sophisticated road networks. The theory about why populated places and transport networks arise at certain geographical locations in the natural environment, and what drives the massive variation in spatial infrastructure are long-standing problems dating from the seminal work of de la Blache^1^. These fundamental questions still attract growing interest nearly a century later, because the resultant geospatial distribution of cities and road networks have major impacts on substantive issues in contemporary society such as urbanisation, traffic congestion, rural depopulation, land use, food supply, and ultimately migration.

In the 1930s and 40s, Central Place Theory emerged as an explanation for the observed hierarchical urban organisation from conceptual models advanced by Christaller^2^ and Lösch^3^, who argued that cities are located hierarchically on a regular 2-D hexagonal lattice as a result of differing population thresholds for viability of particular goods or services, offset against maximum travel distances^4,5^. In the 50s, Isard was instrumental in combining these ideas with earlier work on agricultural land use^6^, and industrial location^7^, in an attempt to construct general equilibrium models within a regional science framework^8^. Further location-theory models were subsequently developed within the New Economic Geography framework, which integrated spatial development into economic models^9,10^, although these also have links back to von Thünen^11^. The critical argument posited by these models is that locations with high or low populations emerge intrinsically without any external environmental differences, with behaviour reminiscent of Turing-type reaction-diffusion pattern formation^12^. Such self-organising spatio-temporal pattern formation is common in both physical^13^ and biological^14^ systems, where structure can spontaneously emerge as a result of the collective behaviour of many agents following simple local rules, without centralized planning or control.

Nevertheless, in these theories, the transport network is typically considered to exist a priori rather than emerge as product of the developing human society. Thus, in most studies, the transport system is treated as an exogenous factor given by external datasets detailing existing road, highway, and railway infrastructure^15–19^. However, in reality, the transport system develops in concert with shifts in human population^20,21^, and iteratively feeds into the overall dynamics of spatial pattern formation, particularly over longer timescales. Furthermore, transport routes are very sensitive to the natural landscape, which is intuitively well recognised, but absent from most models.

Thus, in this paper, we go back to the primary question of the impact of natural landscape on patterns of population, using a model where both cities and roads are allowed to emerge from dynamical Turing-like pattern formation, and the only a priori constraints are based on physical geography. In particular, we consider that mountains, hills, rivers, lakes, and coastlines are likely to have a substantial effect on both the population distribution and the transport network, but are rarely considered quantitatively^19,22^. We capture these elements by inclusion of high-resolution topographic maps to sculpt the surface on which the model unfolds by progressively transforming the underlying space from the typical idealized isotropic ‘flatland’ to the real landscape. The resultant Landscape-Transport-Population (LTP) reinforcement model has three key elements: (i) Detailed landscape topography at 90 m resolution; (ii) The effective transport distance across the real-world landscape; and (iii) Turing-like pattern formation driven by flow-based reinforcement for population distributions. Our approach draws on our previous works^23–27^ where a biologically-inspired, current-reinforcement rule can construct realistic transport networks between food-supply points under physical and physiological constraints, and the principle of co-evolution of nodes and links that dynamically organize scale-free networks via a diffusion process^28,29^.

## Overview of the dynamical model

**Figure 1:**
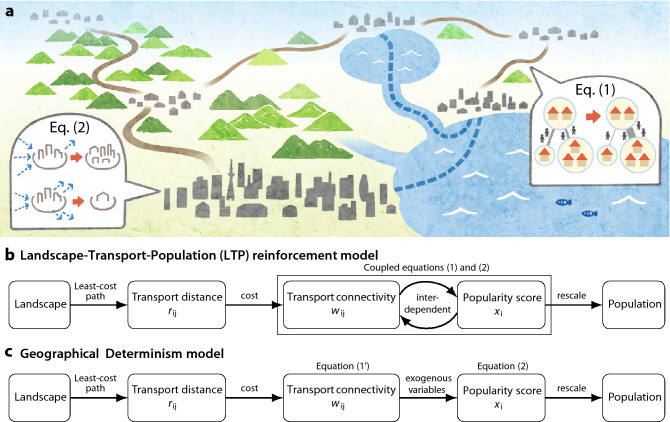
(**a**) Schematic illustration of dynamical pattern formation in the LTP-reinforcement model where populated places and their inter-connections emerge naturally across the landscape. Transport links following least-cost paths across the landscape are strengthened between locations with large populations, whilst well-connected centres in turn tend to grow to a greater extent. (**b,c**) Schematic diagram of the LPT-reinforcement model (**b**) and a counterfactual, ’Geographical Determinism’ model with no reinforcement of the transport network (**c**).

A schematic illustration of how the dynamical model evolves across a landscape is shown in Fig. 1a. Growing centres of population are connected via transport routes across land, rivers, lakes or sea. Preferential transport routes between locations are calculated by gradient-based least-cost path analysis on topographic GIS maps, taking into account the costs of moving over rough terrain or water bodies (see Methods for details). Given the effective transport distance of such a route between each pair of locations, *i* and *j*, we introduce a connectivity variable that weights the amount of traffic and trade. We model development of this transport connectivity using a mass-action formula, which originated from chemical reaction processes and has been applied to other population and social dynamics^30^:

1 $$\begin{aligned} w_{ij}(t+1) -w_{ij}(t) = \epsilon \left[ x_i(t) \cdot x_j(t) - f(r_{ij}) w_{ij}(t) \right] . \end{aligned}$$

Thus the change in transport connectivity is an increasing function of the popularity (normalised population) of the end-point locations given by variables $x_i$ and $x_j$ and offset by the increasing cost of transport with distance. The dependence on distance is given by $f(r) = \frac{r}{R} \exp (r/R)$, where *R* represents the typical length scale of transportation in the epoch under consideration. This specific form of *f*(*r*) originates from the distance deterrence factor in spatial interaction models^31–34^. The Eq. (1) leads a global equilibrium $w^*_{ij} = x_i x_j / f(r)$ when $x_i$ is fixed. $\epsilon$ controls the time-scale of the evolution of this connectivity.

Next, we formulate the coupled hypothesis that centres of population will develop at important transport intersections that reflect the network centrality of each location. Here we use PageRank centrality^35^ as a well-characterised estimator of the relative popularity of location *i*, according to Eq. (2):

2 $$\begin{aligned} x_i(t+1) - x_i(t) = d \sum _{j \in {\mathcal {N}}_i} \left[ T_{ij}(t) x_j(t) - T_{ji}(t) x_i(t) \right] + (1-d)\left( \frac{1}{N} - x_i(t) \right) , \end{aligned}$$

where the transition probability $T_{ij}(t)$ from *j* to *i* is given by $\frac{w_{ij}(t)}{\sum _{k \in {\mathcal {N}}_j} w_{kj}(t)}$ and *N* is the number of locations. The first term sums the incoming and outgoing flows to location *i* from connected locations via the network, giving preference to locations with strong transport connectivity $w_{ij}(t)$. The second term models global dispersion driven by non-network factors, to ensure the agricultural hinterland around the cities remains populated. The balance between these two terms is controlled by parameter *d*. This popularity $x_i$ accumulates the probability of visiting location *i* via a diffusion process on the weighted network, with all probabilities summing to unity. The actual population $X_i$ is determined by scaling this normalised variable by the total population from the census data (see Supplementary Information 3.7 for details).

The initial state of the system variables $x_i,w_{ij}$ is another important factor which determines the final outcome of the simulation. In the following simulation, we firstly do not provides any specific information on the initial state which is set to be nearly homogeneous with small random fluctuations (see Supplementary Information 3.4 for full details). Later, we set the initial state to be similar to the situation in the Roman era and discuss on the effect of the specific initial condition on the temporal evolution of the system.

Figure 1b summarises the coupled system of Eqs. (1) and (2) schematically. The natural landscape is incorporated into the model only through the impact on the least-cost transport distance $r_{ij}$. The resultant transport connectivity $w_{ij}$ then interacts inter-dependently with the popularity at each location $x_i$. In other words, as populated places develop or decay, the connections among them are restructured to meet current needs. This network restructuring causes further expansion or contraction of populated places. Although the model has only three parameters: *R*, $\epsilon$ and *d* (see Supplementary Information 3 for full details), the reinforcement dynamics exhibit rich behaviour.

## Results

### Impact of varying landscape scenarios on population distribution

**Figure 2:**
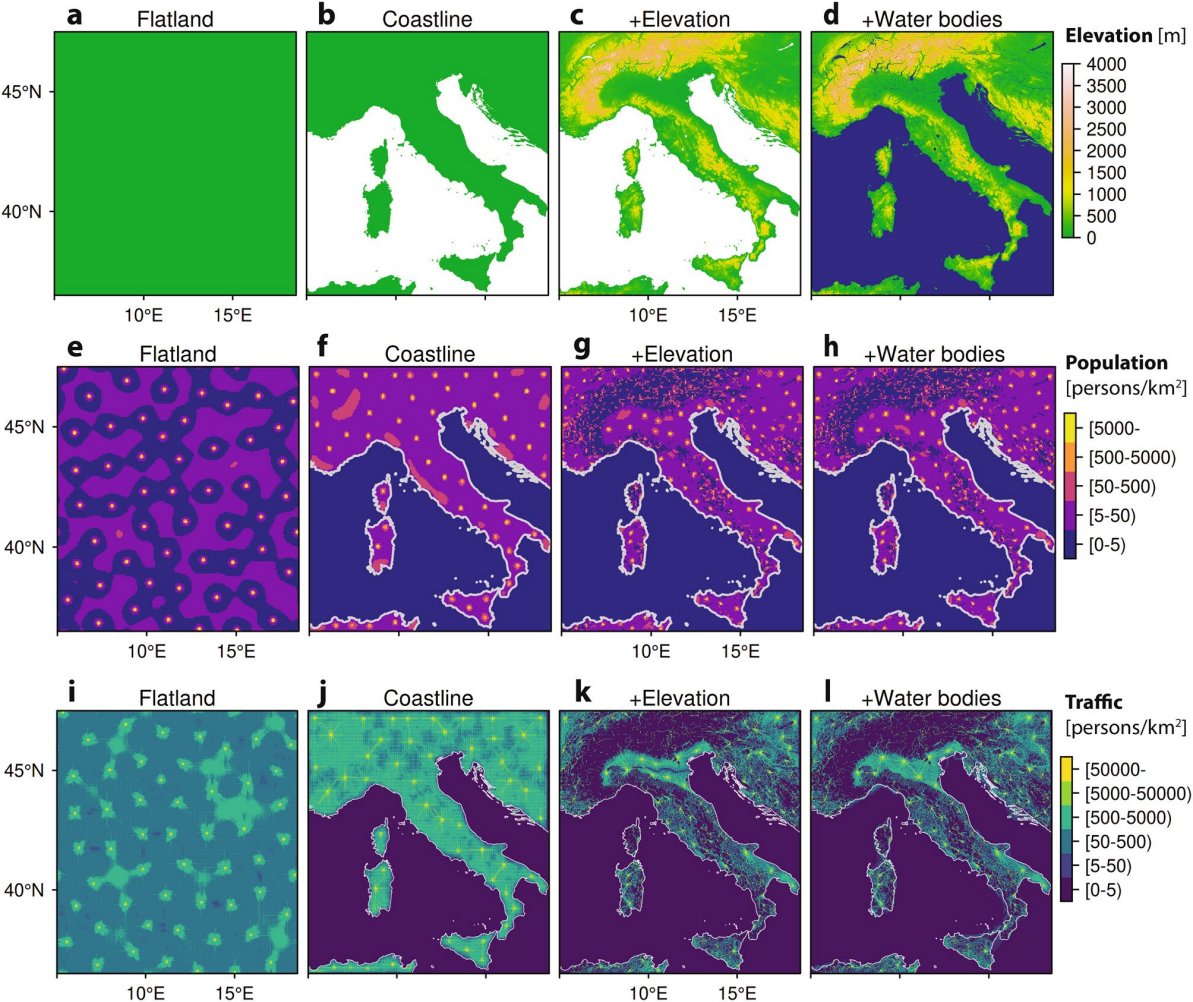
The effect of landscape factors on emergent geospatial patterns of both human population distribution and the interconnecting transport network. The underlying space is changed from an idealised flatland condition (**a**) to a more realistic one by sequentially adding coastlines (**b**), elevation (**c**), and water bodies (**d**), using Italy and its surrounding regions as a case study. By adding these factors, the emergent population pattern produced by the model shifts from a lattice-like Turing pattern with regular population sizes to a markedly heterogeneous distribution (**e–h**). In parallel, the transport network also emerges between centres of population, as depicted by the net traffic ${\hat{W}}(x)$ passing through each location (see Supplementary Information 3.8 for details) (**i–l**).

The impact of landscape factors on the emergent pattern of population distributions and transport networks was explored for four scenarios using Italy as an exemplar. The underlying space was changed from: (i) an idealised isotropic ‘Flatland’, with no geographical features and no boundaries (Fig. 2a); (ii) imposition of the Mediterranean Sea and Italian ‘Coastline’ (Fig. 2b); (iii) inclusion of terrestrial topography to give ‘Elevation’ (Fig. 2c); and (iv) addition of ‘Water Bodies’ such as rivers and lakes which augment the transport possibilities (Fig. 2d). In the isotropic ’Flatland’ scenario the underlying space is uniform and symmetric with periodic boundary conditions. Nevertheless, a regularly-spaced, lattice-like pattern of populated places, with roughly equal population, still spontaneously emerges as a result of the reinforcement dynamics in the model (Fig. 2e). Each populated place also acts as a hub in an almost radially symmetric traffic network (Fig. 2i).

This phenomenon of pattern formation is widely seen in reaction-diffusion systems^36^. Linear stability analysis of the isotropic state reveals that an Eigen mode with a finite wavelength initially becomes unstable as the control parameter *d* is varied above a critical threshold ($d_c$ =0.8), and the mode then develops to give the observed pattern (see Supplementary Information 7 for the details).

By adding landscape factors, the population and transport networks show more complex structure. In the ’Coastline’ scenario, uniformity and symmetry are broken, with the sea providing a major barrier to movement, although the land is still flat. The emergent pattern now deviates from a perfect lattice, but still generates a reasonably regular array of similar sized cities (Fig. 2f), with some preferential strengthening of transport links to nearest neighbours (Fig. 2j). In the Elevation scenario, the least-cost paths for transport are affected by the local gradient of the landscape, and trigger a significant shift away from a regular lattice with substantial variation in population size and location (Fig. 2g). The transport network is also more complex with hubs emanating from the major cities on the northern plane, supplemented by coastal highways and occasional trans-Alpine routes (Fig. 2k). Finally, inclusion of ’Water bodies’ permits transport across rivers, lakes, and seas, which previously acted as barriers in the other scenarios, and opens up the possibility of marine transport along the coast (Fig. 2l), that further modifies the population distribution (Fig. 2h).

### Comparison of the model output with the 2011 census data

**Figure 3:**
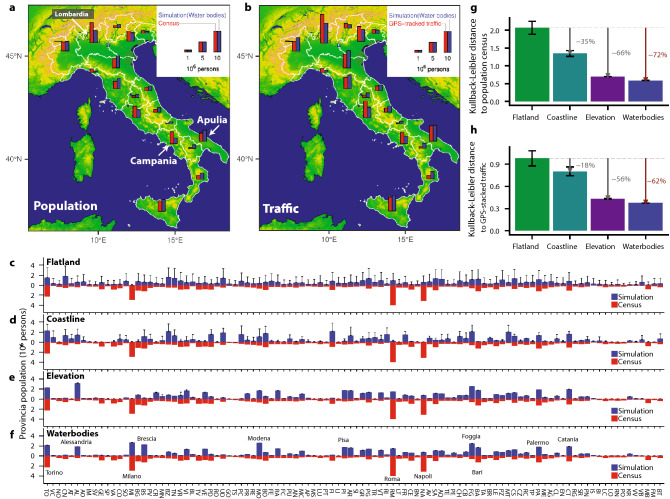
Quantitative comparison between the population and traffic datasets for Italy and the LTP simulation results for different landscape scenarios. (**a**) Population distribution from the 2011 census data for Italy (red) and the LTP simulation (blue) when aggregated at the *Regione* level for the ’Water bodies’ scenario. (**b**) GPS-tracked traffic distribution from OpenStreetMap for Italy (red) and the LTP simulation (blue) when aggregated at the *Regione* level for the ’Water bodies’ scenario. (**c–f**) Comparison in population distribution as in (**a**), but at *Provincia* level for each scenario. Error bars depict the standard deviation that comes from the 64 simulation runs for each scenario. (**g,h**) The Kullback–Leibler (KL) distance from the simulation data to the census population (**g**) and the GPS-tracked traffic datasets (**h**) for *Provincia*-level distributions. The error bars are the same as in (**c–f**).

We then asked whether there are any similarities between the emergent patterns from the model and actual data on populations and transport. Figure 3 shows comparison with the 2011 population census data, and GPS-tracked traffic data at two administrative levels (see Methods for the details of these datasets). The aggregate population and traffic are shown at the *Regione* level in Fig. 3a, b, respectively. There is substantial variation in both the census population and traffic data between regions, but somewhat surprisingly, much of this heterogeneity is faithfully captured by the LTP model, even though the model only has information of the landscape. Nevertheless, the match is not perfect, with the most obvious differences in *Lombardia* and *Campania*, which are under-estimated, and *Apulia* which is over-estimated.

At higher spatial resolution, the population at *Provincia* level is shown in Fig. 3c–f for the four different landscape scenarios. Error bars depict the standard deviation ($n=64$) with different initial conditions (see Supplementary Information 3.4 for details). The Flatland scenario leads to substantially different populations in each *Provincia* at each realisation, apparent from the large error bars, and gives little overall match to the census data (Fig. 3c). Inclusion of more landscape features progressively reduces the variance at each location, stabilises the output, and improves the match between histogram profiles from simulation and census (Fig. 3d–f). Much of this improvement arises from inclusion of elevation (Fig. 3e). However, the additional transport routes available in the ‘Water Bodies’ scenario, allow increased growth of *Milan* (MI), for example (Fig. 3f), and the population of other major cities, such as *Turin* and *Palermo* match the census well. Nevertheless, populations of *Rome* and *Naples* are under-estimated, whilst those in *Alessandria*, *Modena*, *Pisa*, and *Foggia* are over-estimated. The comparison for traffic data at the *Provincia* level also showed a similar improvement with the addition of landscape factors (Figure S5 in Supplementary Information 5).

It is noted that changes in response to the underlying landscape reflect the response of the complete system of cities linked by adaptive connections, not just cities in isolation. For example, a population increase in Naples induces not only population changes in neighboring places, but also the changes in the connections around it. These changes induce further changes at distant locations, and propagate over the entire network in subsequent time steps. Therefore, the effect of the landscape has to be interpreted through its impact on the entire system, not as separate local influence on individual cities.

To evaluate the match between these distributions quantitatively, we used the Kullback–Leibler (KL) distance^37^ from the simulation distribution (*q*) to the census data (*p*), according to Eq. (3):

3 $$\begin{aligned} D_{\mathrm {KL} } (p||q) =\sum _i p(i) \log \left( \frac{p(i)}{q(i) } \right) , \end{aligned}$$

where *p*, *q* are the normalized spatial distribution such that $\sum _i p(i) = \sum _i q(i) = 1$. The index *i* of *p*, *q* indicates the individual *Provincia* or *Regione*, not the size rank. The KL distance progressively decreases as the landscape factors are included for both the population estimates (Fig. 3g) and traffic predictions (Fig. 3h). The Water Bodies scenario gives a total improvement of 72% for population and 62% for traffic. This improvement is also confirmed by other metrics, such as Pearson’s correlation and cosine similarity, which test slightly different aspects of the distributions (see Supplementary Information 6 for the details).

### Counterfactual comparison without dynamic reinforcement

**Figure 4:**
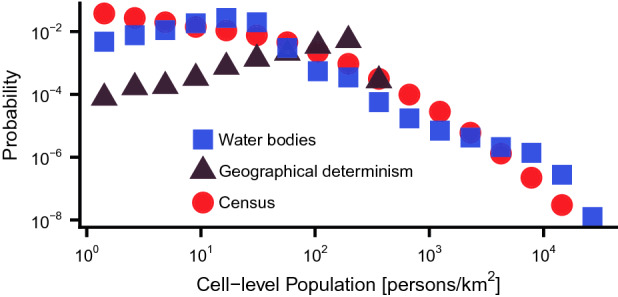
Population density distributions on a  6 km grid showing a typical Zipf’s Law relationship for the census data (red), and the LTP model (blue), which shows a comparably broad distribution. In contrast, a counterfactual ‘Geographical Determinism’ scenario (brown), with the same landscape conditions as ‘Water Bodies’, but lacking the reinforcement dynamics of the model, does not fit the census population data.

One explanation for these results might be that the population pattern simply reflects the external landscape alone—an idea reminiscent of geographical determinism^38,39^, whereby the local population would essentially match the slope metric. However, we argue that the observed pattern could require the reinforcement dynamics linking population aggregation and transport connectivity. To clarify this argument, we introduce a counter-factual scenario termed Geographical Determinism (Fig. 1c). In this scenario, the underlying landscape conditions are the same as the Water Bodies scenario, but with no transport reinforcement dynamics (see Supplementary Information 4 for details). The Geographical Determinism model yields some variation in population size, but only within a narrow range (Fig. 4). In contrast, the LTP simulation show a strongly heterogeneous distribution, in agreement with the census data (Fig. 4). It is noted that this result does not reject geographical determinism in general. Instead, we argue that the reinforcement dynamics provides an alternative mechanism that could be a contributing factor to the heterogeneity.

The fat-tailed distribution of population is already well-known as Zipf’s law (Fig. 4)^40^, and its potential origin extensively investigated^41–44^. The LTP model provides an alternative mechanism that could be a contributing factor to the heterogeneity: we already showed that roughly equal-sized populated places emerge in an idealized ‘flatland’ scenario but shifted to be strongly heterogeneous by inclusion of the underlying real-world landscape in the dynamical model, while the landscape on its own was insufficient. Thus, the combination of the heterogeneity of the underlying landscape and the intrinsic reinforcement dynamics in the model could be a potential driver of a fat-tailed population distribution.

### Inclusion of historical population estimates improves model predictions

**Figure 5:**
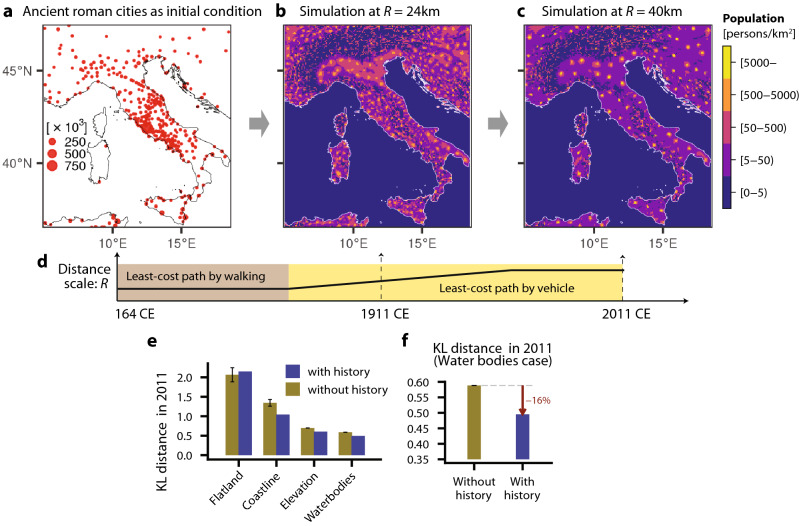
Historical dependency of the simulation on the past population distribution in Italy. (**a–d**) The initial state (**a**) is given by estimates of population distribution in ancient Roman cities. Starting from this initial state, the intermediate state of the simulation at *R* = 24 km (**b**) and the final state with *R* = 40 km (**c**) are depicted, as the distance scale, *R*, is increased ramped up from 8 to 40 km during the simulation (**d**). (**e,f**) Improvement in the KL distance from the 2011 census data at the *Provincia*-level by the inclusion of historical development is shown for all scenarios (**e**), and zoomed-in for the final Water Bodies scenario (**f**).

We then explore the effects of including two elements of “history” in the model. First, we use the population distribution of Roman cities^45,46^ to specify the initial conditions (Fig. 5a). Second, we vary the characteristic transport distance *R* in the cost function (Fig. 5d) to reflect changes in the transport system for each historical age (see Methods and Supplementary Information 1 for details). In the Roman era, the predominant transport modes on land were limited to walking, ox-carts or horse-drawn carriages. We assume that such a transport system has a shorter characteristic distance *R* (= 8 km) than that of a contemporary motorized society (*R* = 40 km), and that sailing is comparably more efficient than movement on land. We therefore follow the evolution of the population with the ramped increase in *R* and the resultant variation in least-cost path parameters (Fig. 5d). At an intermediate state with *R* = 24 km, the model predicts many medium-sized towns and the beginning of larger cities on the northern plain (Fig. 5b). In the final state with *R* = 40 km, some of these populated places merge into several larger metropolises, particularly in north Italy, at the expense of locations that were dominant in previous epochs, but have subsequently declined (Fig. 5c).

**Figure 6:**
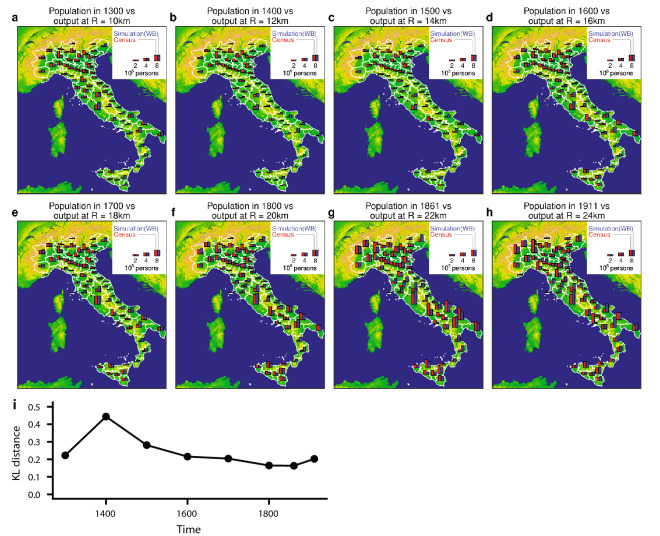
Comparison between the population distribution during the period 1300–1911 and the simulation output from intermediate levels of *R*. (**a–g**) As shown in Fig. 5d, the least-cost distance and characteristic transport distance *R* were varied during the simulation. The intermediate states of the model up to *R* = 22 km were compared with the estimated population distribution from city population data during the period 1300–1861^47^ (see Methods for the details of the estimates). The population distributions from both the simulation output and the estimated data are aggregated by *Provincia* using the 1911 regions, as digital shape data is available at this time point. Red bars indicate the estimated populations and blue bars are the simulation output from the Water Bodies scenario. (**h**) Population distribution at 1911 for Italy (red) and the LTP simulation (blue) at *R* = 24 km when aggregated at *Provincia* level in 1911. (**i**) Quantitative comparison using the KL distance from the model outputs at intermediate values of *R* to empirical data shown in (**a–h**).

Figure 6 compares the intermediate predicted population distributions up to *R* = 24 km with the empirical population distribution estimates from 1300 to 1861^47^ and the census data for 1911. These are aggregated at the *Provincia* level using boundaries existing in 1911 following re-unification, as these are available in digital format for the whole Italian Peninsula. As shown in Fig. 6, much of the spatial heterogeneity is faithfully captured by the model across these ages. The quantitative comparison shows that the KL distance remained around 0.2 during this period (Fig. 6i), with the notable exception of 1400 when city populations were drastically reduced by the plague pandemic.

The effect of including these historical elements was evaluated by the improvement of KL distance (Fig. 5e, f). Compared to initial near-isotropic starting conditions, the constrained historical evolution gave a further improvement of 16%, which provides a quantitative measure of the extent that integration of these historical pathways has had an impact on contemporary society.

## Discussion

Large-scale regional pattern formation of cities and transport networks is a key theme of long-standing importance in the study of human civilization. Here we present a dynamical model in which populated places and their connections co-evolve inter-dependently, and we used this to examine how the emergent patterns are influenced by the detailed topographical landscape. The model incorporates spatial scales from tens of meters (from topographic maps) to thousands of km (the overall modelling domain) and is demonstrated for Italy as a test case. We show that in an idealized ‘flatland’ scenario, a regular pattern of cities with roughly equal population spontaneously emerge, consistent with the arguments set out in Central Place Theory by Lösch^3^, and observed in the initial NEG models^9^. However, inclusion of the natural landscape adds considerably more richness (heterogeneity) to the emergent patterns of both population distribution and the transport network. The resultant patterns are broadly comparable to contemporary population census data and GPS-tracked traffic data, including emergence of a fat-tailed distribution of population density, as seen in the empirical Zipf’s law. We consider it remarkable that such a minimal model can explain complex geospatial patterns not only of population, but also of transport networks. Thus, we would argue that a major contribution of this paper that it reveals the importance of the natural landscape on human civilization with the phenomenological model of co-evolving dynamics of cities and roads, both qualitatively and quantitatively using information-theoretic measures referenced to the actual census data.

To set these results in context, we believe there are several important differences in this modelling approach to those adopted in recent urban economic models. First, our model has extremely few parameters, whose values are set completely independently from the actual population data. In comparison, existing models include detailed micro-economic processes, with the aim of providing as realistic an output as possible to inform policy, planning and investment. This results in elaborate systems with a huge number of location-specific fudge factors^15^, such as attractivity^17^, local amenity^16,48^, or local productivity^22^, which are then tuned by calibration using the actual population data itself.

Second, our model only uses landscape datasets as a priori conditions to reproduce the spatial patterns of population. In contrast, in addition to calibration against the population data itself, alternative realistic models incorporate a wide range of external datasets, including *inter alia* employment levels, economic growth, birth and death rates, bilateral trade flows income and demographic characteristics^15,16,49^. Nevertheless, the natural landscape itself is not included directly in most models, or plays a very limited role. For example, in a few studies landscape factors have been used to validate the calibrated local amenity^48^, as a ruggedness parameter in counterfactual simulations^22^, or to evaluate the local suitability^19^ by elevation or slope in land-use allocation within cellular-automaton based models^18,19^.

Third, in our model, the transport infrastructure is not given a priori, but develops inter-dependently with shifts in population. In existing models, contemporary rail, road and water transport networks are often supplied exogenously and the accessibility of different locations are imposed a priori and only vary to a limited extent, for example when modelling congestion in intra-urban models^19,50,51^. The spatial pattern of these networks already provides a strong guide to where populated places are located. This is important when making short term predictions on local development relevant to policy and investment, but does not address the origin of these networks over longer time periods^17,52^.

In summary, in contrast to existing models, which involve thousands of tunable parameters and detailed external datasets to fit population data as precisely as possible, here we adopted an opposite minimal approach. The system provides an abstract representation that captures the co-evolution rules governing emergence of populated places and their interconnections, when constrained solely by the topographic landscape without any other external datasets. The true population is only used to compare with the output of the model to evaluate its performance, not for calibrations. Thus, our modelling framework provides a straightforward explanation as to why some locations become so populated and others do not, by the direct influence of topographic landscape.

We next show that landscape on its own is not sufficient to explain the population distribution as a form of geographical determinism, but requires the inter-dependent dynamical feedback between population and the transport network emerging in parallel. Such co-evolution has been highlighted as a potential driver of self-organizing system for adaptive biological and social networks^53^, including transportation networks in an idealized city^54^ or region^55,56^. In general, the reinforcement by such adaptive spatial connections represents a macroscopic, ‘rich-gets-richer’ effect in social dynamics. Several positive feedback effects, such as economy of scale, or agglomeration^10^ are well established in spatial economics, whilst other feedback mechanisms have also been incorporated in spatial interaction models^57–59^. In particular, the recursive iteration feedback employed by Wilkinson et al.^60^, embodies remarkably similar principles to the proposed LTP reinforcement model for transport dynamics.

It is notable that de la Blache^1^ also considered the significance of historical factors on the subsequent development of the population distribution, in addition to the central role of the natural landscape. We have begun to explore these possibilities in the model, by incorporating aspects of historical evolution, such as the estimated populations in ancient Roman cities as the starting condition. In the context of dynamical systems theory, the inclusion of historical elements is related to the issue of ‘multistability’: the model has multiple possible equilibrium states as the result of its evolution, whilst there is only one actual realisation in history. In the model, the initial condition determines which one of the possible states is realised in the simulation. In other words, the past condition can affect the future state by selection from multiple possible outcomes. Compared to the average from multiple runs using noisy near-isotropic initial conditions, starting with the “historical” condition from the ancient Roman period selects a better final outcome to the contemporary census dataset in a single model run (Fig. 5).

There are several limitations of the model that need to be considered carefully. The model appears to work well for Italy, which has natural boundaries set by the Mediterranean Sea and the Alps to the North. However, we would expect the population outside the simulation region to influence locations inside, but these are not captured in the scope of the model. Scaling the model up to a continental or global level to avoid these edge artefacts is technically plausible, but currently computationally prohibitive unless one adopts greater constraints on the initial conditions to limit the model runs, or sacrifices some of the spatial resolution to reduce the grid size.

There are also some limitations in the topography dataset, with implications for the predicted impact on transport. Although the GIS data is high-resolution, it does not capture small rivers or narrow passes in mountainous areas that might have a disproportionate impact on transport routes. Furthermore, whilst the coefficients for calculation of the least-cost paths across the landscape are externally derived from other studies, the values are only approximate for each period under study. There are also several coefficients that require refinement, but at the expense of generality. For example, inclusion of larger rivers in the Water Bodies scenario has the positive benefit of permitting river freight and preferential transport between linked river locations. However, as currently implemented, it also uniformly removes the river as a barrier to crossing along its length. In reality, fords and bridges are relatively sparse and have a strong impact on local centres of population, but their location cannot be predicted with the resolution currently available. In addition, the underlying landscapes are assumed to be unchanged in the model, whilst in practice the landscape has been altered by engineering of transport routes to explicitly circumvent barriers, using tunnels, bridges, or elevated roads. Thus, the situation assumed in the model would be more relevant to epochs before the introduction of highly structured and planned transport networks in the modern era, such as motorways, railways and air transport.

Another limitation arising from the assumptions in the simplified model, is that various resources needed to support populations are uniformly distributed. In reality, resources are spatially-varying, and the model can be partly reformatted to include a spatially-varying population capacity, $C_i$ , in Eq. (2) (see Supplementary Information 3). Moreover, as resources are consumable, shortages may arise from large populations, curtailing local growth. The negative feedback by consumable resources will be important to consider for the sustainability of cities and the transport connections between them. In addition, the model is mathematically described as a dynamical system. It assumes deterministic and gradual changes of the system, while rare, but large, interurban migratory shocks might also have a significant effect on long-term growth of cities^44^.

Overall, we argue that the LTP model should probably be best considered as providing a sophisticated null model to facilitate thought experiments about the spatial patterns of cities and roads, and the factors that might influence them. In this framework, the LTP model provides a baseline reference tool to predict the expected population distribution when constrained solely by topography, in the absence of higher-level socio-economic or cultural factors. In this sense, the deviation of the real-world data from the model at each location is perhaps the most interesting feature, as it quantitatively indicates the influence of (untested) spatially-varying environmental factors, such as climate, water, soil, and crop productivity, on the one hand, or local economic, social, cultural, historical and political factors, on the other. For example, the predicted population of Rome is about a third (35.9%) of the actual number, suggesting that its status as both a capital city and a religious centre has stimulated three-fold more growth than expected. To test such factors empirically, the model can be reformatted to include a spatially-varying population capacity or other factors with relevant datasets for future works. This approach provides a systematic way to test the impact of various local or regional factors individually or in combination and how their impact might propagate across the network. We argue that such a bottom-up, building-block approach starting with known external factors drawn from physical geography offers a new direction to deconstruct the complex phenomena of human civilization involving many natural and social factors.

## Methods

### Calculation of transport distances across landscapes

#### Topographic datasets

We used the Shuttle Radar Topography Mission (SRTM) 1 Arc-Second Global elevation dataset for the digital elevation model, and the SRTM Water Body, and Global River Classification (GloRiC) datasets for ocean, lakes and rivers. These datasets are publicly available, distributed by the United States Geological Survey (USGS)^61^ and World Wildlife Fund (WWF)^62,63^. In this study, we used the region ranging from $5^{\circ }$ to $18.55^{\circ }$ longitude, to $36.5^{\circ }$ to $47.5^{\circ }$ latitude that contains Italy and the surrounding Mediterranean Sea. The SRTM datasets contain the information on the type of cell, such as land, ocean, lake, or river, and the elevation in meters for each cell type. The data were downsized to 3 arcsecond spacing by median resampling to reduce the effects of outliers and reduce the computational complexity. To supplement the river network, rivers in the GloRic dataset greater than hydrologic class 1 were included.

#### Least-cost path analysis

We used standard GIS least cost path analysis^64^ to calculate the route for any detour, taking into account the topography. Thus, for each cell in the topographic dataset, the transportation cost to move to its eight adjacent neighbours was evaluated. The cost depends on both the slope, and cell type of the origin and destination cells. The least cost path between any pair of cells was then calculated on the grid graph to minimize the accumulated cost along the overall length of the route, *r*. See Supplementary Information 1 for full details of the calculation and parameters.

### Datasets for comparison with simulation results

#### Census in 2011

The census in 2011 was taken from the GEOSTAT 2011 grid dataset, distributed from Eurostat, EFGS^65^. In Fig. 3, this census data was aggregated by Italian administrative units (*Regione* and *Provincia*). The boundary information of these administrative units in 2011 was obtained from the Italian National Institute of Statistics^66^.

#### Census in 1911

The *Provincia* population in 1911 (Fig. 6) was taken from a data repository^67^, originally from the Almanach de Gotha^68^. The boundary information of *Provincia* in 1911 was obtained from the Italian National Institute of Statistics^66^.

#### Estimated city population sizes in the ancient Roman period (164CE)

The population of the ancient Roman cities shown in Fig. 5a were obtained from the open-access database of the Oxford Roman Economy project^45,69^. The cities that existed at 164CE in the target region were selected from the database. The populations of the cities were estimated using a scaling law between city area and population, as proposed by Hanson and Ortman^46^. The sum of city populations over the cities shown in Fig. 5 was 2,800,832, which is less than the estimated population in the entire area of about 12 million^70^. The urbanisation rate rarely exceeded 20% of total population at this time^71^, thus the difference between these numbers represents the rural population outside the cities in total. The spatial distribution of the rural population is less clear because of methodological difficulties, but it is probably higher close to urban sites in highly urbanized areas, whereas it may be distributed further away from urban sites in low-populated regions^72^. We therefore modelled the spatial distribution of the rural population by a biased distribution to the locations close to populated cities, which is proportional to a distance-discounted score *s*(*i*) at each location *i*:

4 $$\begin{aligned} s(i) = \sum _{u \in \text {ancient roman cities}} \left[ r_{i j_u} / R \right] ^{-1} \times \text {Population({ u})}, \end{aligned}$$

where $j_u$ denotes the location of a city *u* and $r_{i j_u}$ is the least-cost distance specified for walking with *R* = 8 km.

#### Estimated population distribution during 1300–1861

During 1300–1861, the populations in Italian cities with greater than 5000 residents were taken from the Italian Urban Population database^47,73^. The locations of the cites were determined by matching their names with the records in the GEONAMES database^74^. The rural population in total outside the cities was calculated from the urbanisation ratio during the ages^47,75^, and its spatial distribution is determined in the same way to that in 164CE—a distribution proportional to the distance-discounted score to populated cities, given by the Eq. (4) with *R* = 8 km.

In Fig. 6, the population distributions during 1300–1861 were aggregated using the *Provincia* boundaries in 1911 following re-unification. This is because (i) a common agglomeration is required to compare the value of KL distance across the middle ages. (ii) High-resolution shape files for *Provincia* in 1911 are available in digital format from the Italian National Institute of Statistics^66^. (iii) *Provincia* in 1911 covers the whole Italian Peninsula, including Roma, Verona, and Vicenza that were excluded from the *Provincia* dataset in 1861.

#### GPS-tracked traffic

We use the traffic data which is publicly available from OpenStreetMap^76^, as a proxy of transportation volume at each location. We used a set of GPS tracks in Italy, collected up to 2013. A track is an ordered list of GPS points describing a path. We excluded uncorrected data which only comprised a single point, and selected the tracks which had a minimum 1 km trip from a location in Italy. The resultant dataset has 63999 tracks with 90600639 points. The number of these points were counted in cells 45 arcsecond $\times$ 30 arcsecond (about 1 km), to give the traffic data describing the number of people passing through each cell. It is noted that anonymous contributors who uploaded the GPS tracks may not necessarily be representative samples from Italy as a whole. Thus, to correct the sample, counts were weighted by the populations in provinces where the tracks originated.

## Data availability

The topographic datasets and the census data that support the findings of this study are publicly available as noted in Methods.

## Code availability

The simulation code of the model is available in a GitHub repository at https://github.com/TakaakiAokiWork/geodynamics/.

## Supplementary Information

Supplementary Information.
