# Supplementary material for: A model for simulating emergent patterns of cities and roads on real-world landscapes
Source: Sci Rep. 2022 Jun 16;12:10093. doi: 10.1038/s41598-022-13758-1 (PMC9203770; doi:10.1038/s41598-022-13758-1)
Supplement: Supplementary file 1 — Supplementary Information. [file 41598_2022_13758_MOESM1_ESM.pdf]

Supplementary Information for A model for simulating emergent patterns of cities and roads on real-world landscapes

Takaaki Aoki, Naoya Fujiwara, Mark Fricker, and Toshiyuki Nakagaki

Contents

1 Calculation of transport distances across landscapes 1
1.1 Topographic datasets 1
1.2 Least-cost path analysis 2
1.3 Transportation costs for traversing each cell type 2
1.4 Parameter specification for scenarios dominated by walking 3
1.5 Parameter specification for scenarios with motorised vehicles 4
1.6 Defining four scenarios for the underlying space: Flatland, Coastline, Elevation, and Water Bodies 4
2 Datasets for comparison with simulation results 5
2.1 Census in 2011 5
2.2 Census in 1911 5
2.3 Estimated city population sizes in the ancient Roman period (164CE) 5
2.4 Estimated population distribution during 1300 - 1861 5
2.5 GPS-tracked traffic data 6
3 The Landscape-Transport-Population reinforcement model 6
3.1 A reaction-diffusion system on an adaptive network 7
3.2 Coarse-graining: The spatial unit of the system 7
3.3 Using cutoff distances to ensure non-negative connectivity weights 8
3.4 Initial conditions 8
3.5 Numerical simulations 8
3.6 Stationary state 8
3.7 Population estimates from the popularity score x_i 10
3.8 Traffic estimates from the transport connectivity w_{ij} 10
4 A counterfactual model of geographical determinism 10
5 Aggregate traffic at the *Provincia* level 11
6 Validation by other metrics 11
7 Linear stability analysis for the homogeneous solution to the model 12
7.1 Homogeneous solution 12
7.2 Linearized system 14
7.3 Eigenvalue equation for the linearized equation of the homogeneous solution 14
7.4 Notes on the eigenvalue equation 15
7.5 LTP reinforcement model without topographic inhomogeneity 15
7.6 Nearest neighbor coupling case 15
7.7 Next nearest neighbor coupling 16
7.8 Role of model parameters 17
References 17

1 Calculation of transport distances across landscapes

1.1 Topographic datasets

We used the Shuttle Radar Topography Mission (SRTM) 1 Arc-Second Global elevation dataset for the digital elevation model, and the SRTM Water Body, and Global River Classification (GloRiC) datasets for ocean, lakes and rivers. These datasets are publicly available, distributed by the United States Geological Survey (USGS)[1] and World Wildlife Fund (WWF)[2,3]. In this study, we used the region ranging from 5° to 18.55° longitude, to 36.5° to 47.5° latitude that contains Italy and the surrounding Mediterranean Sea. The SRTM datasets contain the information on the type of cell, such as land, ocean, lake, or river, and the elevation in meters for each cell type. The data were downsized to 3 arcsecond spacing by median resampling to reduce the effects of outliers and reduce the computational complexity. To supplement the river network, rivers in the GloRic dataset greater than hydrologic class 1 were included.

1.2 Least-cost path analysis

The topography of the landscape was used to determine the best route between two points, specifically avoiding mountains, lakes, rivers or oceans, where a direct path may be too difficult to follow (see Figure S1).

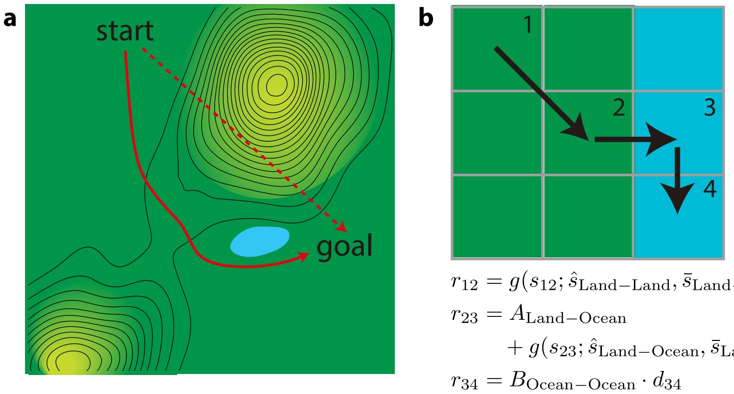

$$r_{12} = g(s_{12}; \hat{s}_{\mathrm{Land-Land}}, \bar{s}_{\mathrm{Land-Land}}) \cdot d_{12}$$
$$r_{23} = A_{\mathrm{Land-Ocean}} + g(s_{23}; \hat{s}_{\mathrm{Land-Ocean}}, \bar{s}_{\mathrm{Land-Ocean}}) \cdot d_{23}$$
$$r_{34} = B_{\mathrm{Ocean-Ocean}} \cdot d_{34}$$

Figure S1. A schematic illustration of the least cost path analysis. (a) Rather than take a direct route (dashed line) over mountainous terrain, for example, we take a detour (solid line) to avoid obstacles in the landscape. (b) The transportation cost r to adjacent cells depends both on the slope s and the cell type (land, ocean, lake or river) of the origin and destination cell. The least cost path minimises the cost along the entire route.

We used standard GIS least cost path analysis[4] to calculate the route for any detour, taking into account the topography. Thus, for each cell in the topographic dataset, the transportation cost to move to its eight adjacent neighbours was evaluated. The cost depends on both the slope, and cell type of the origin and destination cells. The least cost path between any pair of cells was then calculated on the grid graph to minimize the accumulated cost along the overall length of the route, r.

In the following sections, we set out the transportation costs for traversing land, oceans, lakes, and rivers, and transfers between each cell type. Then, we define critical parameters in the equations to reflect the transport costs for two different transport epochs, specifically a historical scenario dominated by walking, and a modern scenario with motorised vehicles.

1.3 Transportation costs for traversing each cell type

First, we formulate the transportation cost for movement across land. Although a number of factors can affect the cost of movement, such as land cover, land use, and climate, the land slope s has been used in most previous studies[4]. From a range of possible slope-based cost functions, we chose a simple quadratic function, with the critical slope $\hat{s}$[5], given by $g(s;\hat{s}) = 1 + \left(\frac{s}{\hat{s}}\right)^2$. This function is more appropriate for wheeled vehicles than other functions[4] and the resulting least-cost paths are in good agreement with actual historical routes when combined with an additional cost component for avoiding streams[6]. The parameter $\hat{s}$ controls the sensitivity to the slope, giving a transition point at which the cost of a straight path on the sloped-hill exceeds a switchback one[5]. If the slope is zero, the cost-based distance equals the direct distance. The cost function g is also symmetric and negative slopes have the same absolute cost to positive ones. As the result, the transport distance between locations was independent of the direction travelled. In other words, the spatial interactions between locations are assumed to be based on round-trip that are accumulated over years, since the time-scale of the simulation runs over millennia, Nevertheless, it should be noted that the model proposed in this paper is not restricted to symmetric distances, but can also accommodate an asymmetric cost function, as the model is defined on a weighted, directed graph.

In addition, we introduced an absolute critical slope parameter $\bar{s}$ that constrains the maximum slope permissible — if the absolute value of slope exceeds this parameter, the cost was set to infinity. As a consequence, the cost-based distance for the route r_{ij} between a pair of adjacent land cells (i,j) is given by:

$$r_{ij} = g(s_{ij};\hat{s},\bar{s}) \cdot d_{ij} = \begin{cases} \left[1 + \left(\frac{s_{ij}}{\hat{s}}\right)^2\right] \cdot d_{ij} & (\|s\| \leq \bar{s}) \\ \infty & (\text{otherwise}) \end{cases}, \tag{S1}$$

where d_{ij} is the direct distance between the cells.

Next, we set out the transportation cost for ships across oceans, rivers or lakes. The empirical cost functions in these situations are not as well characterised as land transportation, so the same quadratic function was used, but with an additional multiplying factor $B > 0$:

$$r_{ij} = B \cdot g(s_{ij};\hat{s},\bar{s}) \cdot d_{ij}. \tag{S2}$$

In practice, the 'slope' was zero for oceans and lakes, but followed the land gradient for rivers. The factor B determines the cost ratio of ship transportation to that on land. The lower the value of B, the more effective transport by ship becomes. This factor B can be different for ocean, river and lake cell types.

Next, we set out the cost function to transition between different cell types. For example, moving from a land cell to an adjacent ocean cell requires transfer to some form of marine transport. To take account of the time penalty associated with transfer, an additive cost $A > 0$ was included:

$$r_{ij} = A + g(s_{ij};\hat{s},\bar{s}) \cdot d_{ij}. \tag{S3}$$

We note that the transit penalty A is defined in units of distance, rather than time.

Finally, the cost functions described above were combined to account for the movement across the four individual types of cells (land, ocean, lake, or river), and the 10 different transfer cases between cell types. These movement costs were described by the same function with different parameter sets, $\hat{s},\bar{s},A$, and B:

$$\begin{aligned}
r_{ij} &= g(s_{ij};\hat{s}_{\text{Land-Land}},\bar{s}_{\text{Land-Land}}) \cdot d_{ij} && (\text{Land}-\text{Land}) \quad \text{(S4)}\\
&= B_{\text{Ocean-Ocean}} \cdot d_{ij} && (\text{Ocean}-\text{Ocean}) \quad \text{(S5)}\\
&= B_{\text{Lake-Lake}} \cdot d_{ij} && (\text{Lake}-\text{Lake}) \quad \text{(S6)}\\
&= B_{\text{River-River}} \cdot g(s_{ij};\hat{s}_{\text{River-River}},\bar{s}_{\text{River-River}}) \cdot d_{ij} && (\text{River}-\text{River}) \quad \text{(S7)}\\
&= A_{\text{Land-Ocean}} + g(s_{ij};\hat{s}_{\text{Land-Ocean}},\bar{s}_{\text{Land-Ocean}}) \cdot d_{ij} && (\text{Land}-\text{Ocean}) \quad \text{(S8)}\\
&= A_{\text{Land-Lake}} + g(s_{ij};\hat{s}_{\text{Land-Lake}},\bar{s}_{\text{Land-Lake}}) \cdot d_{ij} && (\text{Land}-\text{Lake}) \quad \text{(S9)}\\
&= A_{\text{Land-River}} + g(s_{ij};\hat{s}_{\text{Land-River}},\bar{s}_{\text{Land-River}}) \cdot d_{ij} && (\text{Land}-\text{River}) \quad \text{(S10)}\\
&= A_{\text{Ocean-Lake}} + g(s_{ij};\hat{s}_{\text{Ocean-Lake}},\bar{s}_{\text{Ocean-Lake}}) \cdot d_{ij} && (\text{Ocean}-\text{Lake}) \quad \text{(S11)}\\
&= A_{\text{Ocean-River}} + g(s_{ij};\hat{s}_{\text{Ocean-River}},\bar{s}_{\text{Ocean-River}}) \cdot d_{ij} && (\text{Ocean}-\text{River}) \quad \text{(S12)}\\
&= A_{\text{Lake-River}} + g(s_{ij};\hat{s}_{\text{Lake-River}},\bar{s}_{\text{Lake-River}}) \cdot d_{ij} && (\text{Lake}-\text{River}). \quad \text{(S13)}
\end{aligned}$$

The parameters $\hat{s},\bar{s},A,B$ and the typical distance R in the distance-based factor $f(r) = \frac{r}{R}\exp(r/R)$ in equation (1) were adjusted to match the transportation system in the epoch under consideration.

1.4 Parameter specification for scenarios dominated by walking

We assume that movement of population and goods in ancient times was achieved predominantly through walking, or assisted by ox carts and horse-drawn carriages. Therefore, we assume that the typical distance travelled, R, was 8km by considering the distance covered during a trip of several hours using these transportation modes. The critical slope $\hat{s}_{\text{Land-Land}}$ has been estimated previously in the range of 8–12% to match known historical routes[7]. It has been also reported that the slopes of Roman roads may have reached 12% in some exceptional locations[8]. Thus, a value of 12% was used for the critical slope $\hat{s}_{\text{Land-Land}}$ and 18% for the absolute critical slope $\bar{s}_{\text{Land-Land}}$ for this transport epoch.

For transport across water, the cost ratio B relative to transport across land was specified in addition to $\hat{s}$ and $\bar{s}$. This parameter determined the typical distance for movement by ships as R/B. We set B to 0.2 for rivers, lakes, and oceans, which means that the typical distance scale was 40km over water in this epoch, which corresponds to a trip lasting several hours for a ship moving at 4–6 knots. The parameters between river cells, $\hat{s}_{\text{River-River}},\bar{s}_{\text{River-River}}$, were assumed to be the same as that on the adjacent land, since there is little information on the slope-dependency.

For movements between different cell types, we specify a transfer penalty A. At interfaces between land and water the penalty $A_{\text{Land-Ocean}}, A_{\text{Land-Lake}}, A_{\text{Land-River}}$ was assumed to be 1km. With the low value of B described above, these values of A gave a relative advantage to transport by ship in ancient times. On the other hand, the slope-dependent cost at land-water interfaces was assumed to be larger[6], which corresponds to dividing the critical slope by an additional factor. Here, we used a factor of 3, and the modified critical slope $\hat{s}_{\text{Land-River}}, \hat{s}_{\text{Land-Lake}}, \hat{s}_{\text{Land-Ocean}}$ becomes 4%. The absolute slopes $\bar{s}_{\text{Land-River}}, \bar{s}_{\text{Land-Lake}}, \bar{s}_{\text{Land-Ocean}}$ were set as 18% as in the case of land transport. No additional penalties or slope-dependent costs were included for transitions between different types of water bodies, such as a move from a river cell to a ocean cell: $A_{\text{Ocean-Lake}}= A_{\text{Ocean-River}}= A_{\text{Lake-River}} = 0$ and $\hat{s}_{\text{Ocean-Lake}}= \hat{s}_{\text{Ocean-River}}= \hat{s}_{\text{Lake-River}} = \bar{s}_{\text{Ocean-Lake}}= \bar{s}_{\text{Ocean-River}}= \bar{s}_{\text{Lake-River}} = \infty$.

These parameters were determined based on prior knowledge on the ancient era independently from the actual population data. Among the parameters, we focus on the typical distance R, which critically determines the spatial distribution of the population as discussed later in Section 7, and checked whether the chosen value (8km) is optimal or not for minimizing the difference between the simulation outcome and the empirical data. Using the KL distance to evaluate the difference, we calculated the optimal R in the Roman era as shown in Figure S2. The chosen value (8km) is not exactly optimal, but almost close to the minimum.

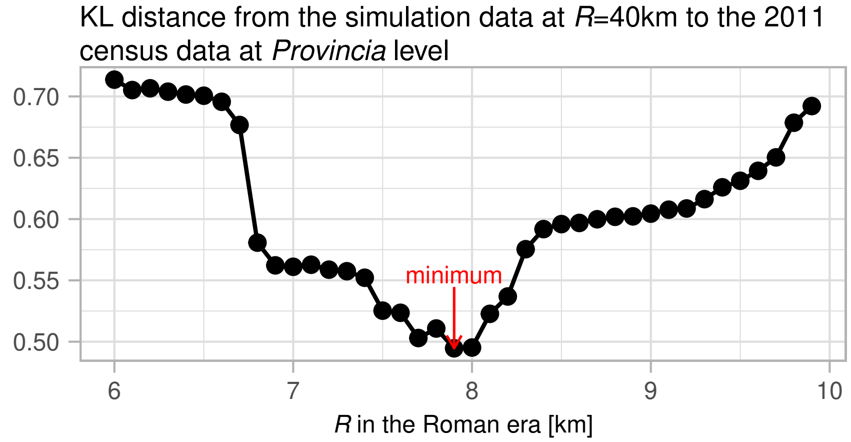

Figure S2. Optimal parameter for R in the Roman era. Starting from the initial state by the estimates of population in the Roman era, we ramped increase in R upto 40km as in Figure 5. The x-axis indicates the initial value of the parameter R. The final simulation state at R = 40km were compared with the census population in 2011 by KL distance (y-axis).

1.5 Parameter specification for scenarios with motorised vehicles

In the modern period, we assume that the major mode of land transport predominantly shifted to automotive vehicles on roads, even though other modes of transportations are also available. The distance scale R on land was set to be 40 km, to reflect a typical one-hour car trip for commuting. The maximum permissible slope for a road was commensurate with the design speed. Thus, in Italy, the maximum slope is in the range of 4–6% for urban streets, and 10% for local roads[9], although steeper roads over these permissible limits still exist. Therefore, a value of 5% was used for the critical slope $\hat{s}_{\text{Land-Land}}$ that served as a soft limit above which the slope significantly affected the transport cost in the quadratic function (S1). The absolute critical slope $\bar{s}_{\text{Land-Land}}$ was set at 12%, which gives an upper limit for the permissible slope.

For transport across oceans and lakes in the modern era, $B_{\text{Ocean-Ocean}}, B_{\text{Lake-Lake}}$ were both set to 0.7, which gives a distance scale for movement by ship of 57.14km, corresponding to a few hours trip at around 20 knots. At 'ports' on the shore, the additive penalties for transfer were given by $A_{\text{Land-Ocean}} = A_{\text{Land-Lake}} = 20$km, corresponding to a loss of half an hour of land transport. The critical slope parameters $\hat{s}_{\text{Land-Lake}}, \hat{s}_{\text{Land-Ocean}}, \bar{s}_{\text{Land-Lake}}, \bar{s}_{\text{Land-Ocean}}$ were set to be the same value as the land layer described above.

We assume that transport across rivers is via bridges and does not involve transfer between vehicles and ships. Thus, the cost ratio $B_{\text{River-River}}$ was set to 1, and no transit penalty was applied ($A_{\text{Land-River}} = 0$). The critical slopes $\hat{s}_{\text{River-River}}, \bar{s}_{\text{River-River}}$ were the same as those on land. However, in a limited number of cases where a path went from land to ocean or lake via a river, transport may be carried by ships. Thus, a transfer penalty for a ship between a river cell and a ocean/lake cell was included, such that $A_{\text{River-Ocean}} = A_{\text{River-Lake}} = 20$km. The other parameters at the interface between different types of water bodies were as follows: $A_{\text{Ocean-Lake}} = 0$ and $\hat{s}_{\text{Ocean-Lake}}= \hat{s}_{\text{Ocean-River}}= \hat{s}_{\text{Lake-River}} = \bar{s}_{\text{Ocean-Lake}}= \bar{s}_{\text{Ocean-River}}= \bar{s}_{\text{Lake-River}} = \infty$.

1.6 Defining four scenarios for the underlying space: Flatland, Coastline, Elevation, and Water Bodies

We considered four scenarios for the underlying space in the model: Flatland, Coastline, Elevation, and Water Bodies. Starting from an idealised isotropic or homogeneous condition, the additional landscape information was integrated into the underlying space in a step-by-step manner. For each scenario, the distances were calculated between each pair of cells by the least-cost path analysis.

The 'Flatland' scenario comprised an idealized isotropic space, where all cells were classed as land and had an identical elevation. To remove the boundary effect of running the simulation in a rectangular space, periodic boundary conditions were used. Furthermore, to follow the traditional assumption of a regular idealized space, this idealized space was approximated as an euclidean space, whilst latitude and longitude co-ordinates are usually defined on an ellipsoid. In the least-cost path analysis, all tiles of 1×1 arcsecond in the dataset were treated as euclidean rectangles, all with the same size defined at the centre tile of the target region.

In the 'Coastline' scenario, ocean cells were included while other cells were land. All land cells maintained the same elevation and no transport across oceans was permitted by setting an infinite penalty for $A_{\mathrm{Land-Ocean}}$.

In the 'Elevation' scenario, the topographic dataset was used without modification, and the slope-based cost for land transportation was included. However, transport on rivers, lakes, and oceans was still prohibited by including an infinite penalty at the transition between these cell types.

In the 'Water Bodies' scenario, transport over rivers, lakes, and oceans were integrated by setting appropriate values of the least-cost path parameters described above. In this scenario, water bodies were no longer barriers, but could act as transport routes between locations if the costs were lower than alternative routes on land.

2 Datasets for comparison with simulation results

2.1 Census in 2011

The census in 2011 was taken from the GEOSTAT 2011 grid dataset, distributed from Eurostat, EFGS[10]. In Fig. 3, this census data was aggregated by Italian administrative units (*Regione* and *Provincia*). The boundary information of these administrative units in 2011 was obtained from the Italian National Institute of Statistics[11].

2.2 Census in 1911

The *Provincia* population in 1911 (Fig. 6) was taken from a data repository[12], originally from the Almanach de Gotha[13]. The boundary information of *Provincia* in 1911 was obtained from the Italian National Institute of Statistics[11].

2.3 Estimated city population sizes in the ancient Roman period (164CE)

The population of the ancient Roman cities shown in Fig. 5a were obtained from the open-access database of the Oxford Roman Economy project[14,15]. The cities that existed at 164CE in the target region were selected from the database. The populations of the cities were estimated using a scaling law between city area and population, as proposed by Hanson and Ortman[16]. The sum of city populations over the cities shown in Fig. 5 was 2,800,832, which is less than the estimated population in the entire area of about 12 million[17]. The urbanisation rate rarely exceeded 20 percent of total population at this time[18], thus the difference between these numbers represents the rural population outside the cities in total. The spatial distribution of the rural population is less clear because of methodological difficulties, but it is probably higher close to urban sites in highly urbanized areas, whereas it may be distributed further away from urban sites in low-populated regions[19]. We therefore modelled the spatial distribution of the rural population by a biased distribution to the locations close to populated cities, which is proportional to a distance-discounted score $s(i)$ at each location i:

$$s(i) = \sum_{u \in \text{ancient roman cities}} [r_{ij_u}/R]^{-1} \times \text{Population}(u), \tag{S14}$$

where j_u denotes the location of a city u and r_{ij_u} is the least-cost distance specified for walking with R = 8km.

2.4 Estimated population distribution during 1300 - 1861

During 1300-1861, the populations in Italian cities with greater than 5000 residents were taken from the Italian Urban Population database[20,21]. The locations of the cities were determined by matching their names with the records in the GEONAMES database[22]. The rural population in total outside the cities was calculated from the urbanisation ratio during the ages[20,23], and its spatial distribution is determined in the same way to that in 164CE– a distribution proportional to the distance-discounted score to populated cities, given by the equation (S14) with R = 8km.

In Fig. 6, the population distributions during 1300-1861 were aggregated using the *Provincia* boundaries in 1911 following re-unification. This is because (i) a common agglomeration is required to compare the value of KL distance across the middle

ages. (ii) High-resolution shape files for *Provincia* in 1911 are available in digital format from the Italian National Institute of Statistics[11]. (iii) *Provincia* in 1911 covers the whole Italian Peninsula, including Roma, Verona, and Vicenza that were excluded from the *Provincia* dataset in 1861.

2.5 GPS-tracked traffic data

The traffic data (Figure S3) is publicly available from OpenStreetMap[24]. We used a set of GPS tracks in Italy, collected upto 2013. A track is an ordered list of GPS points describing a path. We excluded uncorrected data which only comprised a single point, and selected the tracks which had a minimum 1km trip from a location in Italy. The resultant dataset has 63999 tracks with 90600639 points. The number of these points were counted in cells 45 arcsecond $\times$ 30 arcsecond (about 1km), to give the traffic data describing the number of people passing through each cell. It is noted that anonymous contributors who uploaded the GPS tracks may not necessarily be representative samples from Italy as a whole. Thus, to correct the sample, counts were weighted by the populations in 2011 within the provinces where the tracks originated.

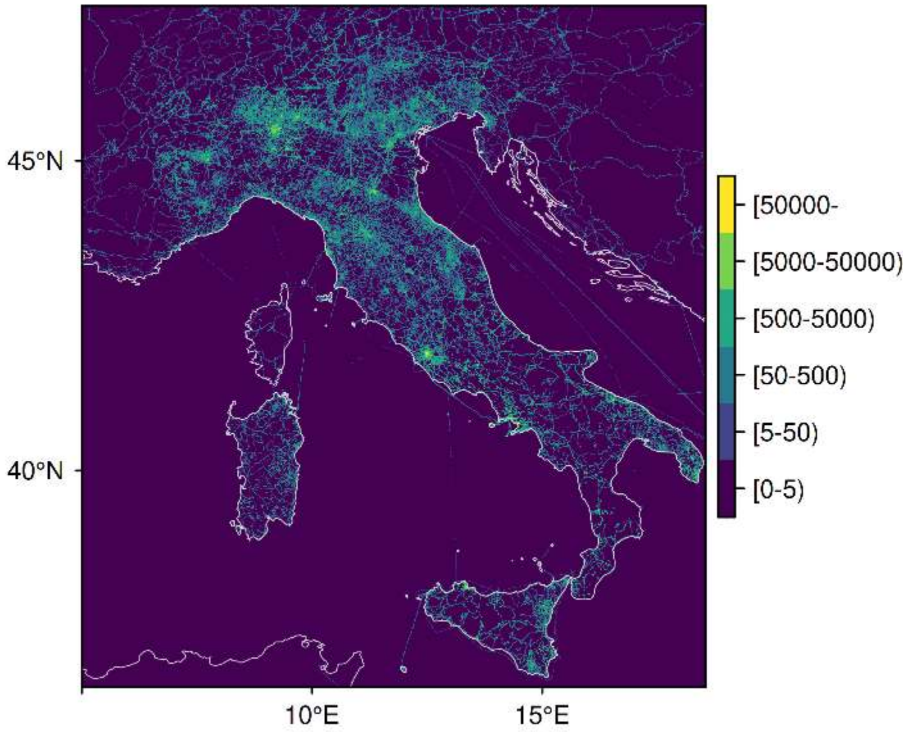

Figure S3. GPS-tracked traffic data, publicly available from OpenStreetMap. We used a set of GPS tracks in Italy, collected upto 2013.

3 The Landscape-Transport-Population reinforcement model

In this section, we present the technical details of the system and numerical simulations.

As described in the main text, the Landscape-Transport-Population (LTP) reinforcement model is defined by equations (1) and (2) as follows:

$$w_{ij}(t+1) - w_{ij}(t) = \varepsilon \left[x_i(t) \cdot x_j(t) - f(r_{ij}) w_{ij}(t)\right], \quad (1)$$

$$x_i(t+1) - x_i(t) = (1-d)\left(\frac{1}{N} - x_i(t)\right) + d \sum_{j \in \mathcal{N}_i} \left[\frac{w_{ij}(t)}{\sum_{k \in \mathcal{N}_j} w_{kj}(t)} x_j(t) - \frac{w_{ji}(t)}{\sum_{k \in \mathcal{N}_i} w_{ki}(t)} x_i(t)\right], \quad (2)$$

where $f(r) = \frac{r}{R}\exp(r/R)$. N is the number of locations (nodes) and the indices i and j range from 1 to N. $\mathcal{N}_i$ is the set of locations that connect to location i. The popularity score $x_i(t)$ and the transport connectivity $w_{ij}(t)$ are the variables of this dynamical system. The parameters ε, d were fixed for all simulations: ε = 0.02, d = 0.81. As described in Section 7, the homogeneous solution was destabilized at d_c = 0.8 without any dependence on ε. Values of d_c just above the critical point were selected. R and r_{ij} were determined by the topography under the given scenario and epoch described in Section 1.

3.1 A reaction-diffusion system on an adaptive network

The dynamical system described by equations (1) and (2) is derived from diffusion dynamics on an adaptive network. Diffusion is a fundamental process associated with many physical and social phenomena operating on real-world networks, and it is considered as a paradigm for dynamical processes on networks. The state of each node at time t is represented by the current fraction of the diffusive population at the node, $x_i(t)$. The network topology with N nodes is given by the adjacency matrix a_{ij}, whilst the link weight from j-th to i-th node for each existing link is denoted by $w_{ij}(t)$. We assume that the population diffuses over the network through the movement of many random agents. Thus the diffusion process is described by:

$$\Delta x_i(t) \equiv x_i(t+1) - x_i(t) = F(x_i(t)) + d \sum_{j \in \mathcal{N}_i} \left[\frac{w_{ij}(t)}{\sum_{k \in \mathcal{N}_j} w_{kj}(t)} x_j(t) - \frac{w_{ji}(t)}{\sum_{k \in \mathcal{N}_i} w_{ki}(t)} x_i(t) \right], \tag{S15}$$

where d is a diffusion constant that controls the strength of diffusion over the whole system. The second and third terms are the inward and outward flows of the population, respectively. The first term $F(x)$ describes a reaction process at the node, which represents the intrinsic dynamics of the population except for the diffusion process. We here assume a simple dissipation process that converges to a capacity $1/N$, given by

$$F(x) = -\kappa(x - 1/N), \tag{S16}$$

where κ is a decay constant. Thus the total popularity score, $\sum_i x_i(t)$, always converges to 1, according to the equation, $\sum_i \Delta x_i(t) = -\kappa(\sum_i x_i(t) - 1)$. This diffusion dynamics can be rewritten as:

$$\Delta \boldsymbol{x}(t) = [\kappa \boldsymbol{G} + d\boldsymbol{T}(t) + (1 - \kappa - d)\boldsymbol{I}]\boldsymbol{x}(t),$$

where $\boldsymbol{x}(t) = \{x_1(t), x_2(t), \cdots, x_N(t)\}$. The elements of $\boldsymbol{G}$, $\boldsymbol{T}$ are $G_{ij} = 1/N$, $T_{ij}(t) = w_{ij}(t)/\sum_{k \in \mathcal{N}_j} w_{kj}(t)$, and $\boldsymbol{I}$ is the identity matrix. If $\kappa + d = 1$, then this equation gives the well-known map iteration (2) that is used to evaluate the PageRank centrality[25].

The dispersion force in equation (2) is given by the dissipation process $F(x)$. In the extreme situation with d = 0, in which all locations are isolated, the population dynamics are governed by $\Delta x_i = F(x_i)$, and the population merely converges to the constant capacity $1/N$. However, if $d > 0$, the population diffuses over the network whose connections are simultaneously reinforced by the local population size. If $d > d_c$, populated places spontaneously emerge on the network as shown in Section 7.

A possible extension of the model is to include space-varying capacity C_i, instead of the constant capacity $1/N$ over all locations as described above. This means that the local dynamics at each node described by equation (S16) can be rewritten as:

$$F(x) = -\kappa(x - C_i). \tag{S17}$$

The equation (1), has a global stable solution under fixed popularity x_i,

$$w_{ij} = \frac{x_i x_j}{f(r_{ij})} = \frac{x_i x_j}{r_{ij}/R} \exp(-r_{ij}/R). \tag{1'}$$

This form is relevant to standard spatial interaction models, such as the "gravity model" and "distance-discounted attractivity", which describes the spatial interactions between two locations by terms of their population and a "deterrence function" that scales with distance r_{ij}. Among the variations of the gravity model, we choose one of the simplest types to remove any additional parameters except R, even though models with more parameters generally give a better match to a given set of data. We combined the form $\frac{x_i x_j}{r_{ij}}$ in the in the early classical papers[26,27], with the additional exponential factor of $\exp(-d_{ij}/R)$, which can represent a critical distance R caused by some physical constraints. It is noted that w_{ij} is scale invariant and only the relative ratio among w_{ij} is important in equation (2). The equation (1) implies that the connections w_{ij} between locations are progressively updated to converge the equilibrium (1'), rather than the connections are instantly built at any moment.

The equations (1) and (2) are well-known, quite simple by themselves: if uncoupled, they have a single global stable solution, and the dynamical behaviour is easily understood. However, if the equations are combined, the co-evolution of the variables $x_i(t)$ and $w_{ij}(t)$ leads to rich phenomena of pattern formation with multi-stability.

3.2 Coarse-graining: The spatial unit of the system

The least-cost distance was evaluated on the high-resolution topographic dataset. However, this spatial resolution is too high to perform numerical simulations. Thus, the LTP model was run on coarse-grained cells of 270 (longitude) $\times$ 180 (latitude) arc-second (about 6.25km $\times$ 5.57km). This gave a total of 39,600 coarse-grained cells in the target region. Thus, each cell had 5,400 location points from the original topographic dataset. If the majority of these points were land (above 50%) the identity of the coarse-grained cell was set to be *land*. Otherwise, it was assumed to be *water*. This gave a total of 18,702 land cells in the target region, where populations could be assigned. Therefore, the total number of locations N in the simulation was 18,702, and indices i and j in equations (1) and (2) run over these locations.

The transport distance r_{ij} between coarse-grained cells i and j was calculated by the least-cost distance between representative points located near the centre of the cell, avoiding water bodies and steep slopes that might increase unnecessary local transportation costs. To be precise, the land point chosen had the minimal slope within the central $1/4 \times 1/4$ of the cell. Figure S4 shows the representative points superimposed on the topographic map.

3.3 Using cutoff distances to ensure non-negative connectivity weights

For an extremely large transport distance r_{ij}, equation (1) potentially leads to negative $w_{ij}(t)$ which is inconsistent with the meaning of the variable. Non-negative values of $w_{ij}(t)$ can be guaranteed by the condition $1-\varepsilon f(r_{ij}) \geq 0$ for positive ε, $x_i(t)$, and $x_j(t)$ with a non-negative initial value $w_{ij}(0)$.

This condition leads to a cutoff distance $r_{ij}^{\max} = 1/f(1/\varepsilon)$. Thus, a set of locations $\mathcal{N}_i$ are connected to location i if the distances to them are below the cutoff. The transport connectivity $w_{ij}(t)$ is defined within the cutoff distance. For any pair of (i, j) beyond the cutoff distance, $w_{ij}(t)$ is undefined, and the locations are assumed to be disconnected. It is noted that because of the exponential term in $f(r)$, the connections beyond the cut-off distance are extremely small (ε order), and negligible. The cutoff distance can be interpreted as the upper limit above which direct transportation is too expensive to maintain, and some stopovers are needed. Owing to the cutoff distance, some cells have no connections to any other cells, and were excluded from the simulations.

It should be noted that all locations interact via the adaptive network even with the cutoff distance. For example, a population increase in Naples induces not only population changes in neighboring places, but also the changes in the connections around it. This induces further re-organization of the adjacent population and traffic flows, which continue to propagate to other locations beyond the cutoff distance.

3.4 Initial conditions

The initial condition of the dynamical system, $\{x_i(0), w_{ij}(0)\}$ for unconstrained scenarios, is given by,

$$x_i(0) = \frac{1}{Z}(1+\xi_i^x), \tag{S18}$$

$$w_{ij}(0) = \xi_{ij}^w, \tag{S19}$$

where Z is a normalized constant to ensure that $\sum x_i(0) = 1$, and ξ_i^x and ξ_{ij}^w are small fluctuations taken from uniform distributions in $[-0.01, 0.01]$ and $(0, 0.01)$, respectively. These small fluctuations are added to avoid the artefactual situation in numerical simulations where the output would otherwise remain in the homogeneous state, and is a standard approach used in studies of pattern formation.

In contrast, the initial condition in the 'historical' scenario (Fig. 5) is unique. In this condition, $x_i(0)$ is given by the estimates of the population in 164CE, normalized so that the sum of them equals 1. $w_{ij}(0)$ is given by the above equation.

3.5 Numerical simulations

The least-cost path analysis and numerical simulations of the equations (1) and (2) were performed using parallel processing with 36 processors.

3.6 Stationary state

By updating the system variables $x_i(t)$ and $w_{ij}(t)$ using equations (1) and (2), the dynamical system converged to a fixed state $\{x_i^*, w_{ij}^*\}$ in all scenarios and all initial conditions that were tested. The convergence was confirmed by observing the following measure at regular intervals, τ:

$$\frac{1}{\tau}\sum_i |x_i(t) - x_i(t-\tau)|\,.$$

The dynamical system given by equations (1) and (2) exhibits multistability. Depending on the initial condition, the system converges to different fixed points.

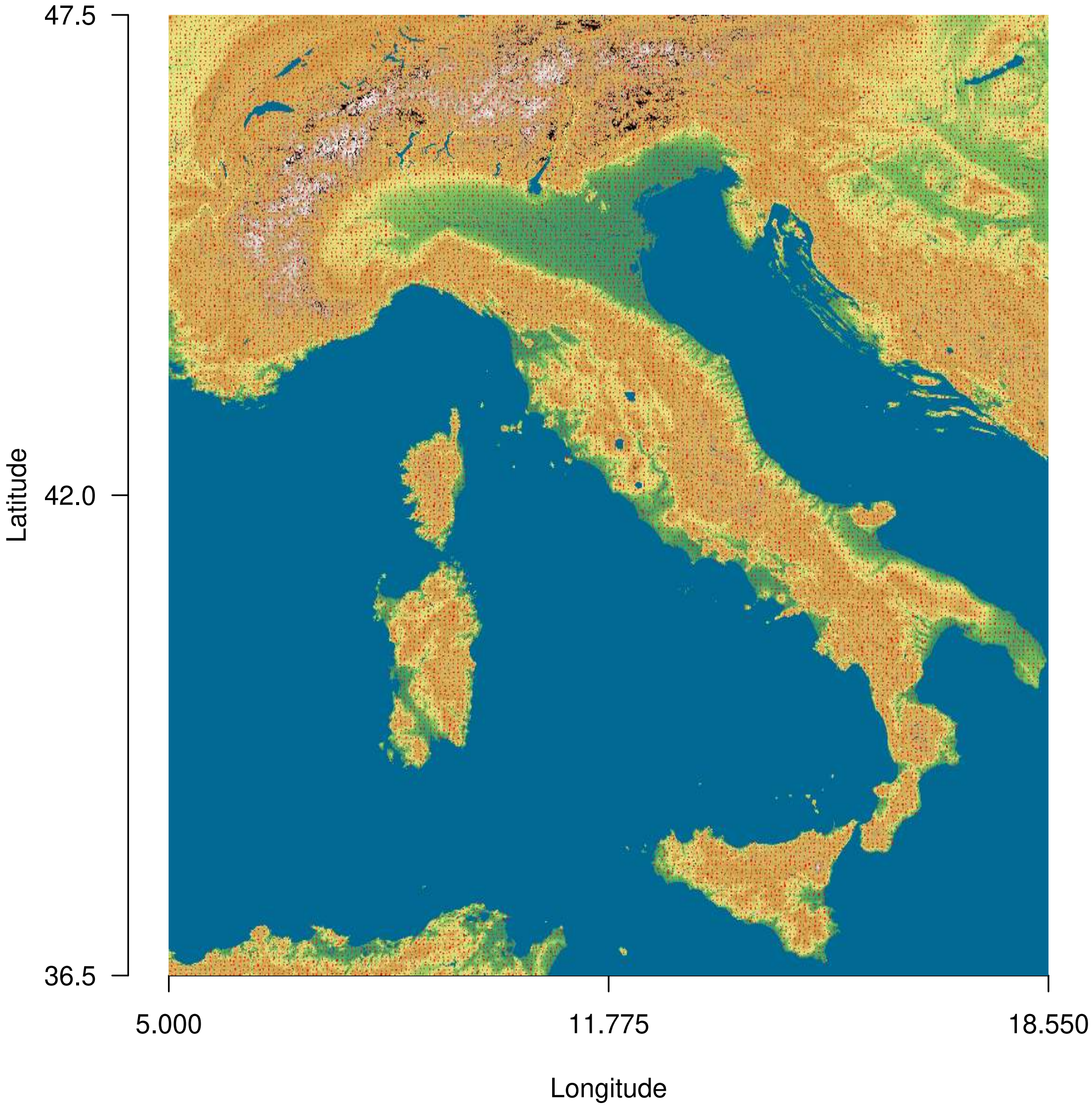

Figure S4. **Topographic map of target region and representative points in each coarse-grained cell.** The target region in this study ranges from 5° to 18.55° longitude, to 36.5° to 47.5° latitude that contains Italy and the surrounding Mediterranean Sea. Shuttle Radar Topography Mission (SRTM) 1 Arc-Second Global elevation dataset were used for the digital elevation model, and the SRTM Water Body dataset and the Global River Classification (GloRiC) dataset for ocean, lakes and rivers (see Section 1.1). The LTP reinforcement model was run on coarse-grained cells of 270 (longitude) $\times$ 180 (latitude) arc-second (about 6.25km $\times$ 5.57km). Red points on the map indicate their representative locations. Transport distance r_{ij} between any pair of the cells are given by the least cost distance between their representative points on the topographic map.

3.7 Population estimates from the popularity score x_i

After obtaining the stationary state $\{x_i^*, w_{ij}^*\}$ for equations (1) and (2), the population X_i at a location i was calculated from the popularity score x_i^* output by the model. The score x_i^* is the probability of random walker being present, with a total summation normalized to 1. To obtain the actual population, the probabilities were rescaled by the total population X_{total} obtained from the census. However, the dispersion term in equation (1), ensured that there was always a tiny positive probability for the presence of a random walker even at tops of mountains in the Alps. Therefore, the minimal score x_{min} was used as the reference for zero population, and the remaining scores scaled so that their summation equalled X_{total}:

$$X_i = X_{\mathrm{total}} \cdot \frac{x_i^* - x_{\mathrm{min}}}{\sum_i (x_i^* - x_{\mathrm{min}})}. \tag{S20}$$

In Fig. 2, the total population X_{total} is given by the summation of the census populations in the depicted region. We note that the census does not cover all countries present in the region, such as North Africa or former Yugoslavia, thus the total population X_{total} is underestimated. In Figs. 3 and 5, where the simulation results are compared to the aggregated populations by administrative units in Italy, X_{total} is the total population of these administrative units in the census.

3.8 Traffic estimates from the transport connectivity w_{ij}

The traffic between locations was calculated from the transport connectivity w_{ij}^* output by the model. In equation (2), the flow from location j to location i per step is given by $\frac{w_{ij}(t)}{\sum_{k\in\mathcal{N}_j} w_{kj}(t)} x_j(t)$. Using the population X_i and X_j described in (S20), the bidirectional traffic W_{ij} between the locations is given by,

$$W_{ij} = \frac{w_{ij}^*}{\sum_{k\in\mathcal{N}_j} w_{kj}^*} X_j + \frac{w_{ji}^*}{\sum_{k\in\mathcal{N}_i} w_{ki}^*} X_i. \tag{S21}$$

The traffic W_{ij} is then assigned to the least-cost path between i and j on the topographic dataset, corresponding to the transport distance r_{ij}. Using a dummy variable $\delta_{ij}(x)$ that returns 1 only if a point x of the topographic dataset is on the path between i and j and otherwise 0, the net traffic $\hat{W}(x)$ passing through each point x was calculated as:

$$\hat{W}(x) = \sum_{i,j} \delta_{ij}(x) W_{ij}. \tag{S22}$$

4 A counterfactual model of geographical determinism

In the main text, a counterfactual model, named *Geographical Determinism* was introduced, whereby the pattern of population was determined directly by the external topographic information, without the internal reinforcement dynamics between populated places and their transport connectivities. We here describe the detailed structure of the model and clarify the differences from the LTP reinforcement model.

In the geographical determinism model, instead of equation (1), the evolution of transport connectivity w_{ij} is described by,

$$w_{ij}(t+1) - w_{ij}(t) = \varepsilon \left[1 - f(r_{ij}) w_{ij}(t)\right], \tag{1'}$$

where the popularity scores at the endpoint locations do not influence the evolution of the weight. This equation has an equilibrium w_{ij}^*,

$$w_{ij}^* = \frac{1}{f(r_{ij})} = \frac{R}{r_{ij}} \exp(-r_{ij}/R). \tag{S23}$$

This result means that the transport connectivity w_{ij}^* is given by the inverse of distance-based cost $f(r_{ij})$. Using this inverse cost w_{ij}^*, we obtain the equilibrium of the popularity score x_i^* from equation (2).

5 Aggregate traffic at the *Provincia* level

The aggregate traffic at the *Provincia* level are shown in Figure S5. The figure depicts quantitative comparison between the traffic dataset for Italy and the LTP simulation results for different landscape scenarios. Error bars depict the standard deviation (n=64) with different initial conditions. Inclusion of more landscape features progressively reduces the variance at each location, stabilises the output, and improves the match between histogram profiles from simulation and traffic data.

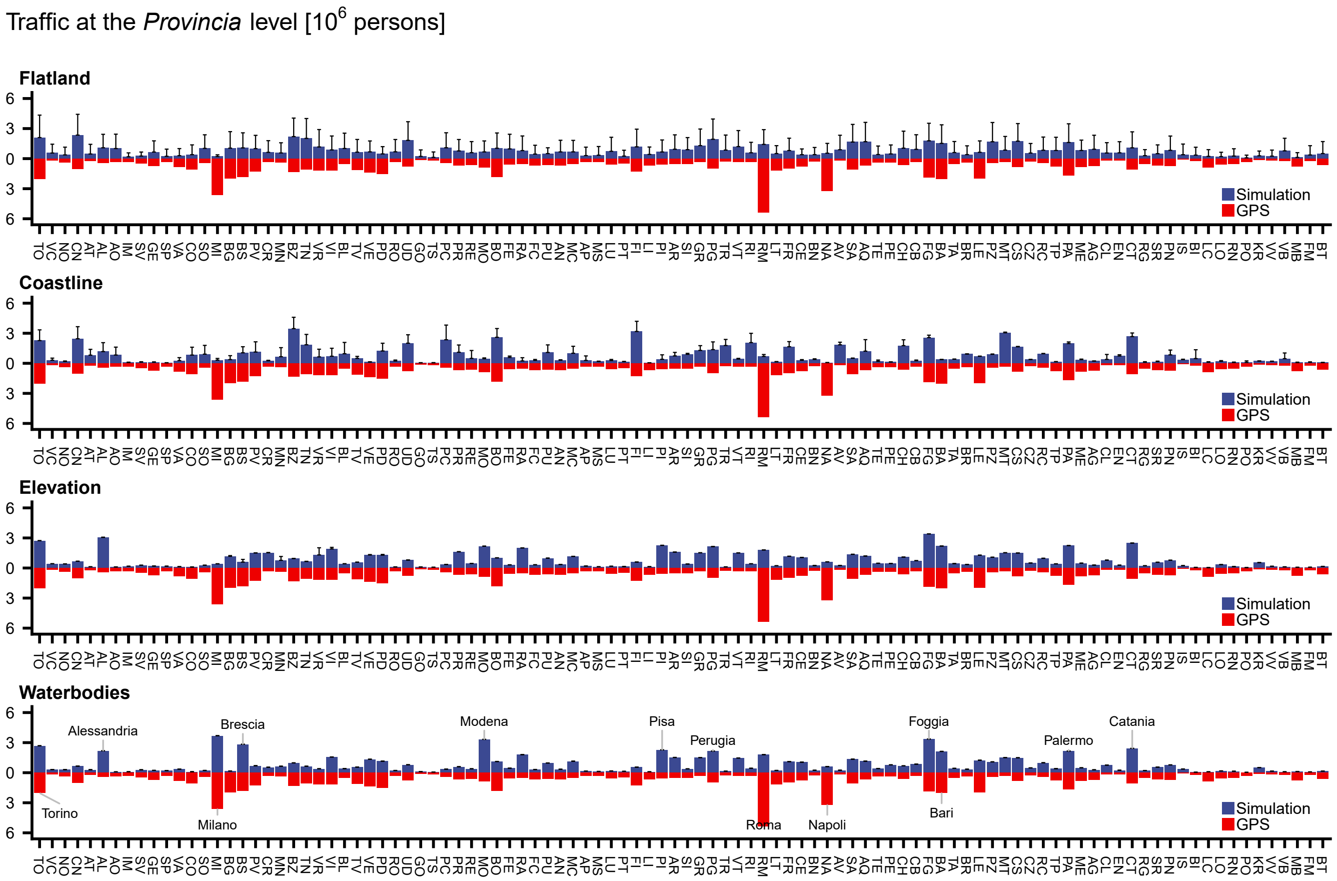

Figure S5. Quantitative comparison between the traffic dataset for Italy and the LTP simulation results for different landscape scenarios. Colored bars show GPS-tracked traffic distribution from OpenStreetMap for Italy (red) and the LTP simulation (blue) when aggregated at the *Provincia* level. Error bars depict the standard deviation that comes from the 64 simulation runs for each scenario.

6 Validation by other metrics

In the main text, we evaluate the difference between the simulation result and the empirical data (Figure 3 **g,h**), and found that the KL distance progressively decreases as the landscape factors are included.

In this section, we validate the improvement by other metrics, namely Pearson's correlation and cosine similarity. These metrics are given by,

$$\text{Pearson's correlation} = \frac{\sum_i (P(i)-\bar{P})(Q(i)-\bar{Q})}{\sqrt{\sum_i (P(i)-\bar{P})^2}\sqrt{\sum_i (Q(i)-\bar{Q})^2}},$$

$$\text{Cosine similarity} = \frac{\sum_i P(i)Q(i)}{\sqrt{\sum_i P(i)^2}\sqrt{\sum_i Q(i)^2}},$$

where P, Q denote the spatial distributions in empirical data (P) and simulation (Q), respectively. The index i of the distributions (P, Q) indicates the individual *Provincia*. $\bar{P}, \bar{Q}$ are the means of the distributions.

Figure S6 shows these metrics for the population distribution for *Provincia*. For the metrics, the inclusion of the landscape factors improves the metrics and reduces the variations among the different initial states. Figure S7 shows the same metrics, but for the traffic distribution, which exhibits a similar improvement to that for the population distribution.

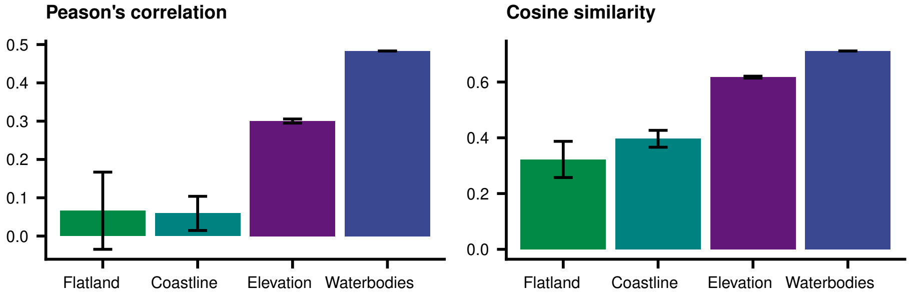

Figure S6. Quantitative comparisons between the census population in Italy 2011 and the LTP simulation results for different landscape scenarios. Each panel shows the different metric to evaluate the difference between the simulation and the census data for *Provincia*-level population distributions. Error bars depict the standard deviation that comes from the 64 simulation runs for each scenario.

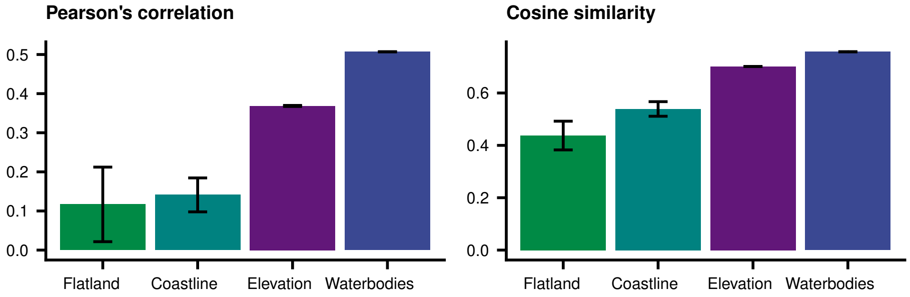

Figure S7. Quantitative comparisons between the GPS-tracked traffic data in Italy and the LTP simulation results for different landscape scenarios. Each panel shows the different metric to evaluate the difference between the simulation and the GPS-tracked traffic data at the *Provincia*-level. Error bars depict the standard deviation that comes from the 64 simulation runs for each scenario.

7 Linear stability analysis for the homogeneous solution to the model

In this Section, we provide a linear stability analysis for the homogeneous solution of the model, to reveal the criteria under which populated places spontaneously emerge.

Figure S8 shows time-sliced images to illustrate the dynamical behavior of the model in the Flatland scenario. Populated places spontaneously emerge as a result of the reinforcement dynamics starting from an almost homogeneous initial distribution, even if there is no topographic inhomogeneity. As shown below, this behaviour is driven by destabilization of the homogeneous solution against a finite wavelength of perturbation, which is a general mechanism of pattern formation.

It should be noted that the model is defined as a network-based system with reinforced connections, not as a classical activator-inhibitor system diffusing in free space. Thus, unlike traditional pattern formation models, the difference in diffusion constants is not the key parameter leading to destabilization in the model. Here we clarify the conditions leading to destabilisation, and discuss the contribution made by the model parameters in this transition.

7.1 Homogeneous solution

Let us consider the homogeneous solution of equations (1) and (2),

$$x_i^* = \frac{1}{N}, \tag{S24}$$

$$w_{ij}^* = \frac{1}{N^2 f(r_{ij})}. \tag{S25}$$

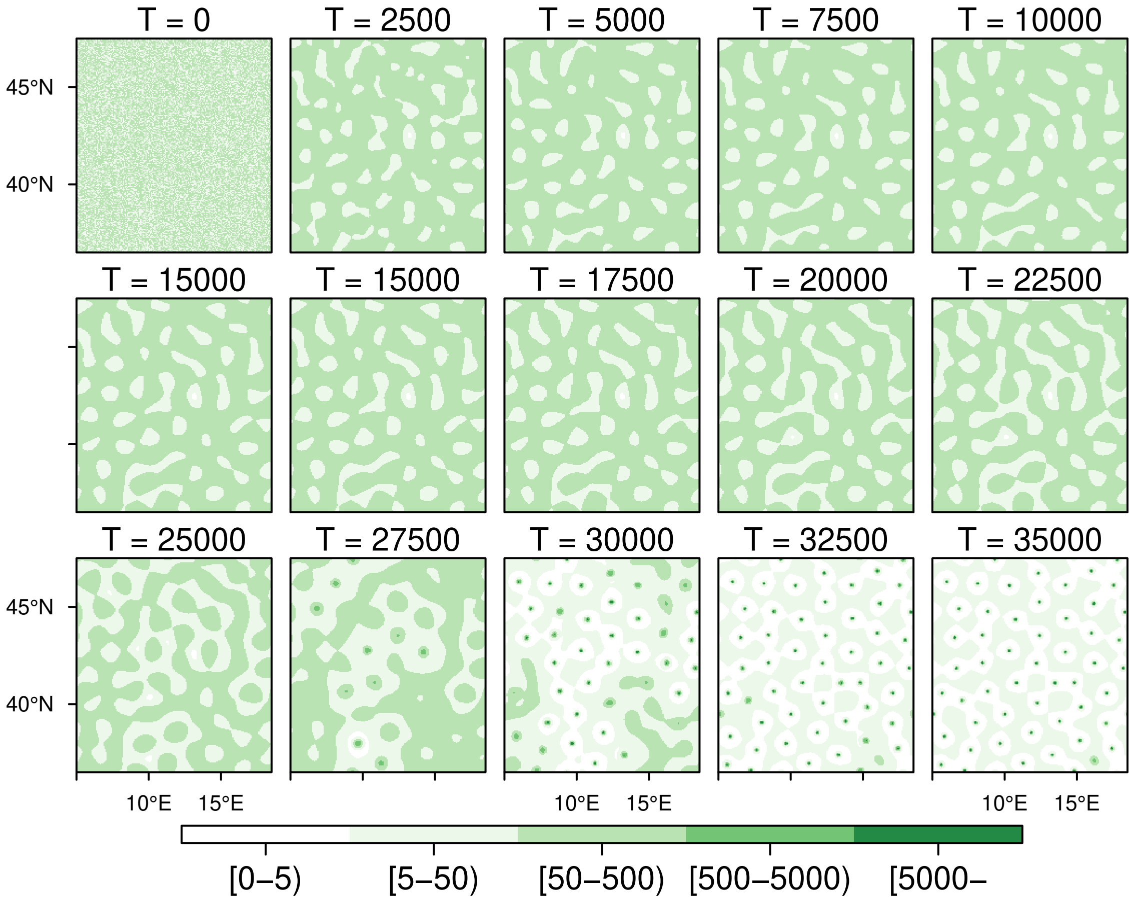

Figure S8. Starting from an almost homogeneous distribution over the land, populated places spontaneously emerge as a result of the reinforcement dynamics in the LTP model.

7.2 Linearized system

First, we derive the linearized equation for $\delta x_i(t) = x_i(t) - x_i^*$, $\delta w_{ij}(t) = w_{ij}(t) - w_{ij}^*$. Substituting equations (S24) and (S25) into equation (2), we obtain

$$\delta x_i(t+1) = \delta x_i(t) + \sum_j J_{ij}\delta x_j(t) + \sum_{j,k} K_{ijk}\delta w_{jk}(t), \tag{S26}$$

where

$$J_{ij} := d\frac{w_{ij}^*}{\sum_k w_{kj}^*} - \delta_{ij} = d\frac{1}{f(r_{ij})C_j} - \delta_{ij}, \tag{S27}$$

$$K_{ijk} := \frac{d}{N}\left[\frac{\delta_{ij}}{\sum_l w_{lk}^*} - \frac{w_{ik}^*}{(\sum_l w_{lk}^*)^2}\right] = \frac{dN}{C_k}\left[\delta_{ij} - \frac{1}{f(r_{ik})C_k}\right], \tag{S28}$$

and $\frac{C_j}{N^2} := \sum_k w_{kj}^* = \frac{1}{N^2}\sum_k \frac{1}{f(r_{kj})}$, and δ_{ij} denotes the Kronecker delta function. It is easy to verify that the linearized equation for (1) is

$$\delta w_{ij}(t+1) = \delta w_{ij}(t) + \varepsilon\left[\frac{\delta x_i(t) + \delta x_j(t)}{N} - f(r_{ij})\delta w_{ij}(t)\right]. \tag{S29}$$

Note that $\sum_i \left[\delta_{ij} - \frac{1}{f(r_{ik})C_k}\right] = 0$ hence $\sum_i K_{ijk} = 0$ holds. Using this equality and taking the summation over i on the both sides of equation (S26), we have $\sum_i \delta x_i(t+1) = d\sum_i \delta x_i(t)$, which means that $\sum_j \delta x_j(t)$ decays in time with the constant rate $0 < d < 1$. Therefore, we can use the initial condition $\sum_j \delta x_j(0) = 0$ without loss of generality.

7.3 Eigenvalue equation for the linearized equation of the homogeneous solution

In the literature on pattern formation, linear stability of the homogeneous solution is usually estimated by analyzing the linear stability of all spatial modes. For systems having spatial translational symmetry, this spatial mode coincides with the spatial Fourier component. However, it is not trivial in the LTP model to know whether we can confirm stability using spatial modes, because some components of the eigenvectors in the Jacobian are labelled by two locations, i.e., w_{ij} has two suffixes i and j.

In order to clarify and solve this problem, let us introduce an $N(N+1)$-dimensional vector

$$\boldsymbol{v} = (\delta x_1, \delta x_2, \cdots, \delta x_N, \delta w_{11}, \delta w_{12}, \cdots, \delta w_{1N}, \delta w_{21}, \cdots, \delta w_{2N}, \delta w_{31}, \cdots, \delta w_{NN})^T, \tag{S30}$$

namely,

$$v_i = \begin{cases} \delta x_i, & 1 \le i \le N \\ \delta w_{\lfloor (i-1)/N \rfloor, (i-1 \mod N)+1}, & N+1 \le i \le N(N+1), \end{cases} \tag{S31}$$

where $\lfloor x \rfloor$ gives the largest integer not larger than x. Then equations (S26) and (S29) can be summarized by

$$\boldsymbol{v}(t+1) = \boldsymbol{T}\boldsymbol{v}(t), \tag{S32}$$

where $\boldsymbol{T}$ is the Jacobian matrix with the size $N(N+1) \times N(N+1)$. If $\boldsymbol{T}$ is diagonalizable, $\boldsymbol{v}(t)$ can be written by the superposition of the eigenvectors of $\boldsymbol{T}$, as

$$\boldsymbol{v}(t) = \sum_i a = 1^{N(N+1)} c_a \Lambda_a^t \boldsymbol{\phi}_a, \tag{S33}$$

where

$$\boldsymbol{\phi}_a = (y_1^{(a)}, y_2^{(a)}, \cdots, y_N^{(a)}, z_{11}^{(a)}, z_{12}^{(a)}, \cdots, z_{1N}^{(a)}, z_{21}^{(a)}, \cdots, z_{2N}^{(a)}, z_{31}^{(a)}, \cdots, z_{NN}^{(a)})^T \tag{S34}$$

is ath eigenvector of $\boldsymbol{T}$. Here we denote the components of $\boldsymbol{\phi}_a$ using $y_i^{(a)}$ and $z_{ij}^{(a)}$. The corresponding eigenvalue is Λ_a, and c_a is a constant determined by the initial conditions.

In order to represent the eigenvectors using quantities only allocated to a single site, let us eliminate $z_{ij}^{(a)}$ from the linearized equations, and rewrite them with $y_i^{(a)}$ only. Since $\boldsymbol{\phi}_a$ is an eigenvector, its component y_i and z_{ij} terms must satisfy the linearized equation (S29) by replacing δx_i and δw_{ij} with y_i and z_{ij}. Recalling equation (S33), we have

$$\Lambda_a^{t+1} z_{ij}^{(a)} = \Lambda_a^t z_{ij}^{(a)} + \varepsilon \Lambda_a^t \left[\frac{y_i^{(a)} + y_j^{(a)}}{N} - f(r_{ij}) z_{ij}^{(a)} \right], \tag{S35}$$

which leads us to

$$z_{ij}^{(a)} = \varepsilon \frac{y_i^{(a)} + y_j^{(a)}}{N[\Lambda_a - 1 + \varepsilon f(r_{ij})]}. \tag{S36}$$

The above equation allows us to convert $z_{ij}^{(a)}$ into $y_i^{(a)}$ and $y_j^{(a)}$, and the components of $\boldsymbol{\phi}_a$ can be represented using terms only containing $y_i^{(a)}$. In this way, we can investigate the linear stability of this system on a spatial coordinate system.

Finally, substituting equation (S36) into equation (S26), we can rewrite the eigenvalue equation for Λ in terms of $\boldsymbol{y}$ as

$$\Lambda y_i = \sum_{j=1}^{N} H_{ij}(\Lambda) y_j, \tag{S37}$$

where

$$H_{ij}(\Lambda) = \delta_{ij} + J_{ij} + \varepsilon \sum_k \frac{K_{ijk} + K_{ikj}}{N[\Lambda - 1 + \varepsilon f(r_{jk})]}. \tag{S38}$$

7.4 Notes on the eigenvalue equation

Note that the right hand side of equation (S37) depends on the eigenvalue Λ. This is different from the eigenvalue equation derived in conventional pattern formation models without adaptive interactions. From this equation Λ can be determined in a self-consistent manner. First we fix Λ and solve the eigenvalue equation. If the eigenvalue obtained coincides with Λ, then Λ satisfies equation (S37). There can be N different terms for a fixed Λ in the denominator of the right hand side of equation (S38). Thus this equation may have Λ^{N+1} terms after reducing the common denominators. By solving this equation with respect to Λ, we may have $N+1$ solutions. Therefore, there can be $N(N+1)$ different eigenvalues in this equation with N variables, and this number is consistent with the size of the $\boldsymbol{T}$ matrix.

In this model, from equation (S29), the transition probability has an equilibrium $\delta w_{ij}(t) = \dfrac{\delta x_i(t) + \delta x_j(t)}{N f(r_{ij})}$ in the linear approximation. For $\varepsilon \to \infty$, this equilibrium is achieved immediately when x_i changes. This can be obtained from equation (S36) by taking the limit $\varepsilon \to \infty$. This result is reasonable, because ε determines the relaxation time scale of w. In other words, the terms $\Lambda_a - 1$ in the denominator of equation (S36) represent the effect of slow relaxation of w.

7.5 LTP reinforcement model without topographic inhomogeneity

Let us consider a specific case, where N sites align on a one-dimensional ring. We further assume no topographic inhomogeneity, i.e. a flat landscape. This means we assume translational symmetry of the system, $C_j = C$ holds for all j, and J_{ij} and K_{ijk} reduce to

$$J_{ij} = \frac{d}{f(r_{ij})C} - \delta_{ij}, \tag{S39}$$

$$K_{ijk} = \frac{dN}{C} \left[\delta_{ij} - \frac{1}{f(r_{ik})C} \right]. \tag{S40}$$

The distance r_{ij} between points i and j ($i \le j$) is given by $r_{ij} = \min(j-i, N+i-j)$. Let us discuss the linear stability of specific cases to see how instability of the homogeneous state takes place.

7.6 Nearest neighbor coupling case

For the simplest case, let us assume that only the nearest neighbors are coupled, i.e., $f(r_{ij}) = 1$ for $j = i \pm 1$ and $f(r_{ij}) = \infty$ otherwise. In this case, $\dfrac{1}{f(r_{ij})} = \delta_{i,j+1} + \delta_{i,j-1}$, $C = \sum_k \dfrac{1}{f(r_{kj})} = 2$, and $K_{ikj} = \dfrac{dN}{2} \left[\delta_{ik} - \dfrac{\delta_{i,j+1} + \delta_{i,j-1}}{2} \right]$ hold. Therefore, we

have

$$J_{ij} = \frac{d}{2}(\delta_{i,j+1} + \delta_{i,j-1}) - \delta_{ij}, \tag{S41}$$

$$\begin{aligned} H_{ij}(\Lambda) &= \delta_{ij} + J_{ij} + \varepsilon \sum_k \frac{K_{ijk} + K_{ikj}}{N(\Lambda - 1 + \varepsilon f(r_{jk}))} \\ &= \frac{d}{2}(\delta_{i,j+1} + \delta_{i,j-1}) + \frac{\varepsilon d}{2(\Lambda - 1 + \varepsilon)} \left[\delta_{ij} - \frac{\delta_{i,j+2} + \delta_{i,j-2}}{2} \right]. \end{aligned} \tag{S42}$$

It is noted that H_{ij} does not depend on R, owing to the assumption of nearest-neighbor coupling. The matrix H is circulant and symmetric. Hence, its eigenvalues are real and can be represented by using the matrix elements as

$$\Lambda_\kappa = H_{ii} + 2H_{i,i+1} \cos(2\pi\kappa) + 2H_{i,i+2} \cos(4\pi\kappa) = \frac{\varepsilon d}{2(\Lambda_\kappa - 1 + \varepsilon)} [1 - \cos(4\pi\kappa)] + d \cos(2\pi\kappa). \tag{S43}$$

where $\kappa = 0, \frac{1}{N}, \frac{2}{N}, \cdots, \frac{N-1}{N}$. The associated eigenvectors are

$$\boldsymbol{y}_\kappa = \frac{1}{\sqrt{N}}(1, e^{2\pi \mathrm{i}\kappa}, e^{4\pi \mathrm{i}\kappa}, \cdots, e^{2(N-1)\pi \mathrm{i}\kappa}) \tag{S44}$$

and its complex conjugate, where $\mathrm{i} = \sqrt{-1}$. Here $N\kappa$ gives the wave number of the eigenvector. In the continuum limit $N \to \infty$, κ can take $0 \le \kappa < 1$. Because of the symmetry, we focus on the range $0 \le \kappa < 0.5$.

Although the eigenvalue Λ_κ can be explicitly obtained by solving the quadratic equation (S43) with respect to Λ_κ, we can estimate the instability of the homogeneous state more easily using the fact that the eigenvalues are real. Thus the instability occurs by crossing either $\Lambda_\kappa = 1$ or $\Lambda_\kappa = -1$. Let us concentrate on the former case. Let $\Lambda_\kappa = 1$, Eq. (S43) can be rewritten as

$$1 = d \left[\frac{1}{2} + \cos(2\pi\kappa) - \frac{1}{2}\cos(4\pi\kappa) \right] =: d \cdot \mathscr{F}(\kappa). \tag{S45}$$

It can be easily verified from the fact that the peak of $\mathscr{F}(\kappa)$ is $5/4$ for $\kappa = 1/6$. Therefore, the mode with $\kappa = 1/6$ becomes unstable at $d = 4/5$ for the first time when d is increased.

7.7 Next nearest neighbor coupling

To discuss the effect of parameter R, which was removed by the assumption of nearest-neighbor coupling in the previous section, we next consider the case that each node is coupled with both the nearest neighbors and the next nearest neighbors. The coupling is given as $f(r_{ij}) = \frac{1}{R}\exp(\frac{1}{R})$ for $j = i \pm 1$, $\frac{2}{R}\exp(\frac{2}{R})$ for $j = i \pm 2$, and $f(r_{ij}) = \infty$ otherwise.

Let us focus on calculating the bifurcation point where the homogeneous state loses linear stability. As in the nearest neighbor coupling case, elements of the H matrix for $\Lambda = 1$, where the instability occurs, are obtained as

$$\begin{aligned} J_{ij} &= d \frac{1}{f(r_{ij})C} - \delta_{ij} \\ &= d \frac{2(\delta_{i,j+1} + \delta_{i,j-1}) + e^{-\frac{1}{R}}(\delta_{i,j+2} + \delta_{i,j-2})}{2(2 + e^{-\frac{1}{R}})} - \delta_{ij}, \end{aligned} \tag{S46}$$

$$\begin{aligned} H_{ij}(1) &= \delta_{ij} + J_{ij} + \sum_k \frac{K_{ijk} + K_{ikj}}{N f(r_{jk})} \\ &= \frac{d}{4(2 + e^{-\frac{1}{R}})^2} \Big[-e^{-\frac{2}{R}}\delta_{i,j-4} - 4e^{-\frac{1}{R}}\delta_{i,j-3} + 2(-2 + 2e^{-\frac{1}{R}} + e^{-\frac{2}{R}})\delta_{i,j-2} + 8\delta_{i,j-1} \\ &\quad + 2(4 + 8e^{-\frac{1}{R}} + e^{-\frac{2}{R}})\delta_{i,j} + 8\delta_{i,j+1} + 2(-2 + 2e^{-\frac{1}{R}} + e^{-\frac{2}{R}})\delta_{i,j+2} - 4e^{-\frac{1}{R}}\delta_{i,j+3} - e^{-\frac{2}{R}}\delta_{i,j+4} \Big]. \end{aligned} \tag{S47}$$

Note that this matrix is identical to the one for the nearest neighbor case for the limit $R \to 0$.

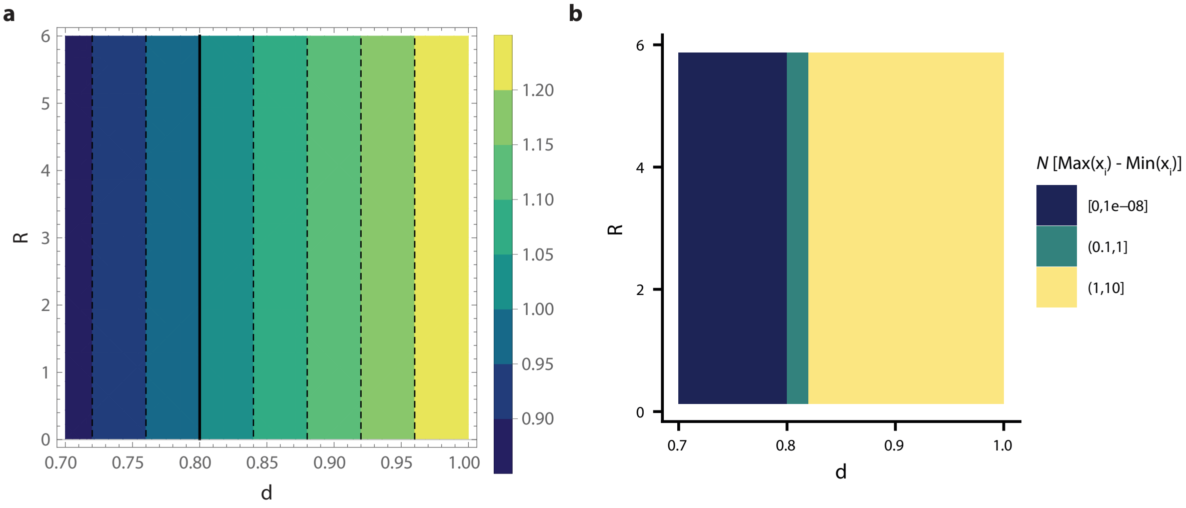

Figure S9. Stable and unstable areas of the homogeneous solution in (R,d) parameter space. **a,** The maximum value $\max_\kappa \mathscr{F}(\kappa;R,d)$ of equation (S48) in (R,d). $\max_\kappa \mathscr{F}(\kappa;R,d) = 1$ occurs at $d = 0.8$, and leads to the destabilisation of the homogeneous solution with eigenvalue $\Lambda = 1$. **b,** Numerical evaluation by $N \cdot [\max_i x_i(t) - \min_i x_i(t)]$ at the stationary state, which should be zero if the homogeneous solution is stable, with $N = 48$.

Finally, at the critical point with eigenvalue $\Lambda = 1$, the relationship between the wave number κ of the perturbation in Fourier mode, and the parameters d and R are given as follows:

$$
\begin{aligned}
1 &= H_{ii} + 2H_{i,i+1}\cos(2\pi\kappa) + 2H_{i,i+2}\cos(4\pi\kappa) + 2H_{i,i+3}\cos(6\pi\kappa) + 2H_{i,i+4}\cos(8\pi\kappa) \\
&= \frac{d}{4(2+e^{-\frac{1}{R}})^2}\Big[2(4+8e^{-\frac{1}{R}}+e^{-\frac{2}{R}}) + 16\cos(2\pi\kappa) + 4(-2+2e^{-\frac{1}{R}}+e^{-\frac{2}{R}})\cos(4\pi\kappa) \\
&\quad - 8e^{-\frac{1}{R}}\cos(6\pi\kappa) - 2e^{-\frac{2}{R}}\cos(8\pi\kappa)\Big] =: \mathscr{F}(\kappa;R,d).
\end{aligned}
\tag{S48}
$$

7.8 Role of model parameters

The model without topographic inhomogeneity has only three parameters: the typical distance scale R, the diffusion constant d, and the time-scale ratio between the two dynamical processes ε. Using the result of the linear stability analysis given by equation (S48), we clarify the role that these parameters play in destabilization of the homogeneous solution.

First, the parameter ε does not appear in equation (S48). Thus, ε is not involved in this type of destabilization.

Second, the diffusion constant d controls the destabilization that triggers spontaneous emergence of populated places. Figure S9a shows the stable and unstable area in (R,d) parameter space, evaluated from equation (S48). This analytical result is numerically confirmed in Figure S9b, in which we plot the value, $N \cdot [\max_i x_i(t) - \min_i x_i(t)]$ at the stationary state. This value should be zero if the homogeneous solution is stable. The boundary between stable and unstable regions depends only on the diffusion constant d, and not on R. By increasing d, $\mathscr{F}(\kappa;R,d)$ increases and hits the line of $\mathscr{F}(\kappa;R,d) = 1$ at the critical point $d_c = 0.8$, as shown in Figure S10a. Figure S10b,c show the behaviour of the model just below or above the critical point, supporting the analytical prediction.

Third, the typical distance scale, R, controls the wavelength of the destabilized Fourier mode at the critical point d_c, but R does not itself trigger destabilization as shown above. At the critical point d_c, the eigenvalue $\Lambda = 1$ at a finite wave number κ_c of the perturbation in Fourier mode. This means that the unstable mode κ_c starts to separate from the homogeneous solution, and leads to the emergence of populated places. Thus, κ_c determines the number of populated places in the finite space (N locations). In other words, it controls the interval distance between the populated places. By increasing R, the unstable mode κ_c decreases, as shown in Figure S11a. This corresponds to fewer populated places with longer distances between them. The numerical simulations support this analytical prediction (Figure S11b).

Thus the parameters of the proposed LTP model have different effects on the pattern formation of populated places that quite distinct and intuitively understandable: (1) The time-scale ratio ε is unrelated to the destabilization; (2) The diffusion constant d determines the destabilization condition $d_c = 0.8$; (3) The typical distance scale R determines the spacing between populated places at the destabilization point.

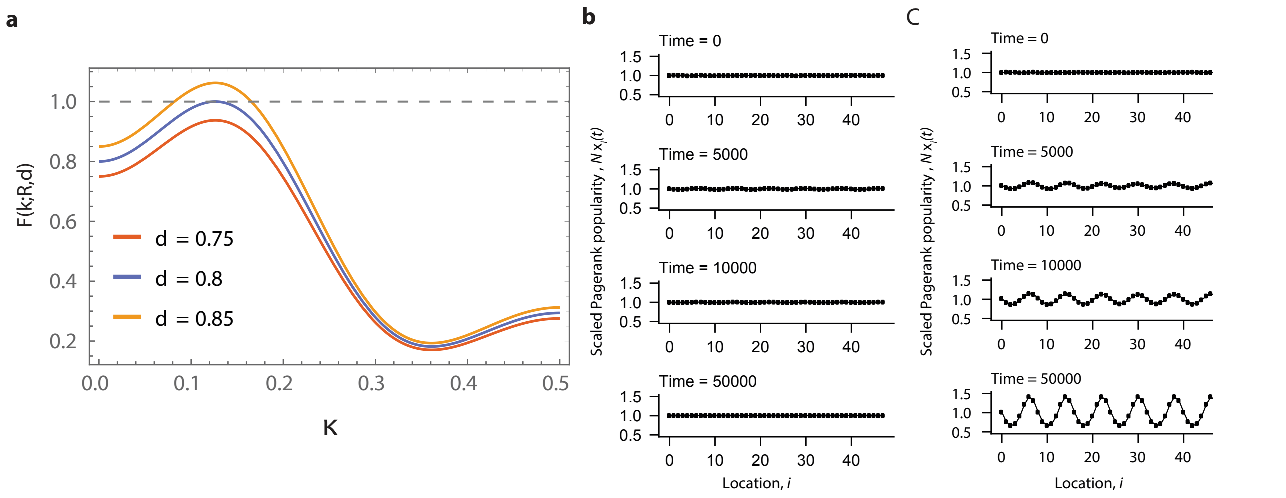

Figure S10. The diffusion constant d controls the destabilization leading to spontaneous emergence of populated places. **a,** $\mathscr{F}(\kappa;R,d)$ of equation (S48) with $R=4$. $\mathscr{F}(\kappa;R,d)$ hits the line of $\mathscr{F}(\kappa;R,d)=1$ at a critical point $d_c=0.8$ with a finite κ_c. **b,** Numerical simulation just below the critical point. $d=0.79<d_c(=0.8)$. **c,** Numerical simulation just above the critical point. $d=0.81>d_c(=0.8)$.

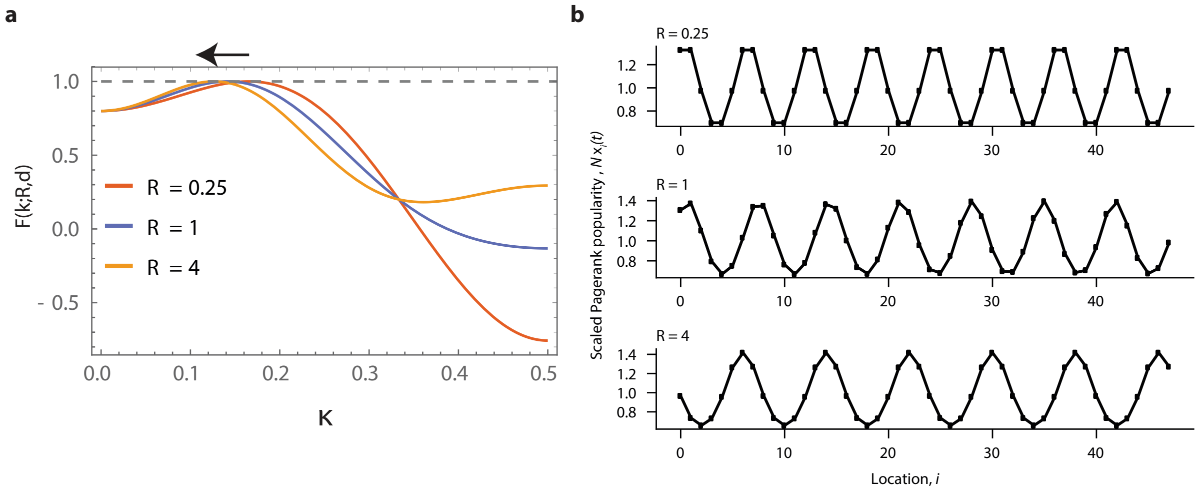

Figure S11. The typical distance scale, R, controls the wavelength of the destabilized Fourier mode at the critical point d_c. **a,** $\mathscr{F}(\kappa;R,d)$ of equation (S48) at $d_c=0.8$. By increasing R, the unstable mode κ_c decreases, leading to longer distances between populated places. **b,** Numerical simulations with changing R.

References

1. USGS. Digital elevation - shuttle radar topography mission (srtm) 1 arc-second global. https://doi.org/10.5066/F7PR7TFT (2015).
2. Dallaire, C. O. & Lehner, B. Global river classification gloric version 1.0. https://www.hydrosheds.org/page/gloric (2018).
3. Dallaire, C. O., Lehner, B., Sayre, R. & Thieme, M. A multidisciplinary framework to derive global river reach classifications at high spatial resolution. *Environ. Res. Lett.* **14**, 024003 (2019).
4. Herzog, I. A review of case studies in archaeological least-cost analysis. *Archeol. e Calcolatori* **25**, 223–239 (2014).
5. Llobera, M. & Sluckin, T. J. Zigzagging: Theoretical insights on climbing strategies. *J. Theor. Biol.* **249**, 206–217 (2007).
6. Herzog, I. Theory and practice of cost functions. In F. Contreras, F. J. M., M. Farjas (ed.) *Proceedings of the 38th Annual Conference on Computer Applications and Quantitative Methods in Archaeology*, vol. 2494, 375–382 (Oxford, Archaeopress, 2013).
7. Herzog, I. & Posluschny, A. Tilt – slope-dependent least cost path calculations revisited. In Jerem, F. R., E. & Szeverényi, V. (eds.) *On the Road to Reconstructing the Past. Computer Applications and Quantitative Methods in Archaeology (CAA). Proceedings of the 36th International Conference, Budapest, April 2-6, 2008*, 212–218 (Archeaeolingua, Budapest, 2011).
8. GREWE, K. Alle wege fiühren nach rom –römerstraßen im rheinland und anderswo. In Koschik, H. (ed.) *Alle Wege führen nach Rom, Internationales Römerstraß enkolloquium Bonn*, vol. 16 of *Materialien zur Bo dendenkmalpflege im Rheinland*, 9–42 (Rhein-Eifel-Mosel Verlag, 2004).
9. Romani, L. Istituto della enciclopedia italiana: Strada. http://www.treccani.it/enciclopedia/strada/, Retr. Sep. 1, 2018.
10. Eurostat. Geostat 2011 grid dataset v2.0.1. https://ec.europa.eu/eurostat/web/gisco/geodata/reference-data/population-distribution-demography.
11. The Italian National Institute of Statistics. Boundaries of administrative units for statistical purposes. https://www.istat.it/it/archivio/222527, Retr. Jan. 8, 2020.
12. Lahmeyer, J. Italy: historical demographical data of the administrative division. http://www.populstat.info/Europe/italyp.htm, Retr. July, 20, 2019.
13. Perthes, J. *Almanach de Gotha* (Gotha, Germany, 1838-1944).
14. Hanson, J. W. *An Urban Geography of The Roman World, 100 BC to AD 300* (Archaeopress, 2016).
15. Hanson, J. W. Cities database (oxrep databases), version 1.0. https://doi.org/10.5287/bodleian:eqapevAn8 (2016).
16. Hanson, J. W. & Ortman, S. G. A systematic method for estimating the populations of Greek and Roman settlements. *J. Roman Archaeol.* **30**, 299–322 (2017).
17. Scheidel, W. Demography. In Scheidel, W., Morris, I. & Saller, R. P. (eds.) *The Cambridge Economic History of the Greco-Roman World*, 38–86 (Cambridge University Press, 2007).
18. de Ligt, L. *Peasants, Citizens and Soldiers: Studies in the Demographic History of Roman Italy 225 BC–AD 100* (Cambridge University Press, 2012).
19. de Haas, T. The geography of Roman Italy and its implications for the development and integration of rural economies. In de Haas, T. C. A. & Tol, G. (eds.) *The Economic Integration of Roman Italy*, 51–82 (Brill, 2017).
20. Malanima, P. Italian Cities 1300-1800. A Quantitative Approach. *Rivista di Storia Eonomica* **14**, 91–125 (1998).
21. Malanima, P. Italian urban population 1300 - 1861 (database). https://www.cnr.it/en/institutes-databases/database/324/italian-urban-population-1300-1861 (2005).
22. Geonames. Geonames. https://www.geonames.org/, Retr. Aug. 25, 2020.
23. Bosker, M., Brakman, S., Garretsen, H., de Jong, H. & Schramm, M. The Development of Cities in Italy: 1300 - 1861. *CESifo* **1893**, 1–43 (2007).
24. OpenStreetMap. Openstreetmap gps data. https://planet.openstreetmap.org/gps/, Retr. Nov. 11, 2020.
25. Page, L., Brin, S., Motwani, R. & Winograd, T. The PageRank citation ranking: Bringing order to the web. Tech. Rep. 1999-66, Stanford InfoLab (1999).
26. Zipf, G. K. The P1 P2/D Hypothesis: On the Intercity Movement of Persons. *Am. Sociol. Rev.* **11**, 677–686 (1946).
27. Ullman, E. L. The role of transportation and the bases for interaction. In Thomas, W. L. (ed.) *Man's Role Chang. Face Earth*, 862–880 (University of Chicago Press, 1956).
